# A central hub for convergence: multi-layer genomic networks reveal lineage-specific plasmid compatibility driving hypervirulence and carbapenem resistance in ST11 *Klebsiella pneumoniae*

**DOI:** 10.3389/fmicb.2026.1932987

**Published:** 2026-09-30

**Authors:** Xuan Hai, Jun-Ling Wang, Qi Chen, Yi-Fei Lu, Xiu-Ping Li, Cong-Jian Li, Chao-Fu Yue, Jian-Mei Song, Ling-Ling Yang, Min Chen, Hong-Wei Li, Xiao-Yang Zhi

**Affiliations:** 1 Yunnan Institute of Microbiology, Key Laboratory of Microbial Diversity in Southwest China of Ministry of Education, School of Life Sciences, Yunnan University, Kunming, China; 2 Center of Clinical Laboratory, Qujing Central Hospital, Qujing Affiliated Hospital of Kunming Medical University, Qujing, China

**Keywords:** core genome synteny, hypervirulence and resistance convergence, *Klebsiella pneumoniae*, multi-layer genomic networks, sequence similarity network

## Abstract

The convergence of hypervirulence and carbapenem resistance in *Klebsiella pneumoniae* represents a critical threat to global public health. However, the genomic mechanisms underlying this convergence, particularly the interplay between chromosomal background and mobile genetic elements, remain incompletely understood. Here, we integrated complete genome data from 102 clinical isolates from Qujing, China, with a global dataset of 4,262 genomes to construct a multi-layer population genomic framework. Using comparative genomics and sequence-similarity network analyses, we characterized the structural organization of the core genome and the population structures of plasmids and prophages. We show that the ST11 lineage serves as a central hub for the convergence of hypervirulence and carbapenem resistance. Plasmids are organized into discrete, functionally specialized communities, among which a dominant community is strongly enriched for hypervirulence and multidrug resistance determinants and preferentially associates with ST11. In contrast, the core genome exhibits a highly constrained structural organization, with most core genes embedded within conserved synteny modules, indicating strong evolutionary constraints on chromosomal architecture. Furthermore, prophages form structured yet more conserved communities that show non-random associations with plasmid types and selectively contribute to the dissemination of resistance genes. Notably, we identified a previously unreported plasmid harboring multiple copies of key virulence loci, underscoring the ongoing modular evolution of high-risk elements. Together, these findings reveal that the convergence of hypervirulence and resistance is driven by lineage-specific plasmid compatibility within a structurally constrained chromosomal background, and they establish a network-based framework for understanding the evolution of high-risk *K. pneumoniae* lineages.

## Introduction

*Klebsiella pneumoniae* is a major opportunistic pathogen responsible for a wide spectrum of healthcare-associated and community-acquired infections (Martin and Bachman, 2018). In recent decades, the rapid global spread of carbapenem-resistant *K. pneumoniae* (CRKp) has severely limited treatment options and posed a major clinical challenge. In parallel, hypervirulent *K. pneumoniae* (HvKp), characterized by enhanced siderophore production and increased invasive potential, has emerged as a cause of severe infections in otherwise healthy individuals. The recent convergence of these two phenotypes—carbapenem resistance and hypervirulence—has led to the emergence of hypervirulent carbapenem-resistant *K. pneumoniae* (Hv-CRKp), representing one of the most concerning evolutionary trajectories among bacterial pathogens (Van Duin and Doi, 2017; Choby et al., 2020; Xie et al., 2021). The emergence of Hv-CRKp is largely driven by horizontal gene transfer via plasmids co-harboring carbapenemase genes (e.g., *bla*_KPC_, *bla*_NDM_, and *bla*_OXA_) and virulence loci (*iucABC*-*iutA* and *rmpA*/*rmpA2*) (Doi, 2019; Russo and Marr, 2019; Lan et al., 2021). Certain high-risk lineages, including ST11 in East Asia and ST258/ST512 in Europe and North America, have played dominant roles in the global spread of carbapenem resistance (David et al., 2019; Hu et al., 2024). More recently, ST11 has increasingly been associated with the acquisition of hypervirulence determinants, suggesting that specific chromosomal backgrounds may be particularly permissive to the convergence of resistance and virulence (Wyres and Holt, 2016; Wang et al., 2024). However, the mechanisms underlying this lineage-specific convergence remain poorly understood.

Understanding *K. pneumoniae* evolution requires bridging different layers of genome organization. While gene content variation across its conserved core and variable accessory genome is well documented (Holt et al., 2015; Wyres et al., 2020), the structural organization of the core genome, specifically whether core genes form conserved syntenic modules, remains systematically unexamined at a species-wide scale. Concurrently, plasmids, as the primary drivers of rapid adaptation, exhibit mosaic architectures poorly captured by traditional replicon typing. Sequence similarity network approaches can resolve plasmids into discrete evolutionary clusters (Redondo-Salvo et al., 2020; Li et al., 2024); however, a global, network-based characterization of the *K. pneumoniae* plasmid population and its alignment with host lineages remains lacking. Furthermore, while prophages also contribute to genome diversification, how plasmids and prophages jointly structure bacterial populations to shape the dissemination of high-risk traits remains poorly understood (Pfeifer and Rocha, 2024).

To address these gaps, we conducted an integrated population genomic analysis that combined regional surveillance data from Qujing, China, with a global collection of complete *K. pneumoniae* genomes. Using a multi-layer framework that integrates core genome analysis with sequence similarity networks of plasmids and prophages, we aimed to resolve how genome structure and mobile genetic elements jointly shape the convergence of hypervirulence and carbapenem resistance. Through this multi-layer approach, we mapped the connectivity between host lineages and plasmid communities, identifying the ST11 lineage as a central hub for acquiring plasmids co-carrying virulence and resistance determinants within a structurally constrained core genome. By integrating these plasmid, core-genome, and prophage dynamics, this study proposes a lineage-specific plasmid compatibility model to explain the emergence and global spread of high-risk *K. pneumoniae* clones.

## Materials and methods

### Bacterial isolates and whole-genome sequencing

A total of 102 clinical isolates of *Klebsiella pneumoniae* were collected from routine diagnostic specimens submitted to Qujing Central Hospital in Yunnan Province, China, between August 2024 and December 2025. These strains were isolated from clinical specimens, including sputum, bronchoalveolar lavage fluid, blood, urine, and drainage fluid. Species identification was performed using matrix-assisted laser desorption/ionization time-of-flight mass spectrometry (MALDI-TOF MS; VITEK MS, bioMérieux, France). Antimicrobial susceptibility testing (AST) was performed using the VITEK 2 Compact system (bioMérieux, France), and results were interpreted according to Clinical and Laboratory Standards Institute (CLSI) guidelines. Whole-genome sequencing was performed by Magigene Biotechnology Co., Ltd. (Guangzhou, China) using a hybrid strategy that combined Oxford Nanopore Technologies (ONT) PromethION long-read sequencing with Illumina NovaSeq PE150 short-read sequencing. Base calling for Nanopore reads was performed using Dorado v0.9.0,^1^ followed by quality filtering. Illumina sequencing produced 150-bp paired-end reads with a Q30 > 90% across all samples.

### Genome assembly, quality control, and annotation

Hybrid assembly was performed with Unicycler v0.4.8 (Wick et al., 2017), which combines long reads for initial scaffolding and short reads for error correction and gap closure, yielding complete circular chromosomes and plasmids. Genome completeness and contamination were assessed using CheckM v1.1.0 (Chklovski et al., 2023). Genomes meeting the thresholds of completeness ≥95% and contamination ≤5% were retained for downstream analysis. Species confirmation was performed using FastANI v1.34 (Jain et al., 2018) against the type strain of *K. pneumoniae* (PartO-Kpneumoniae-RM8376 = ATCC 13883^T^; GCF_022869665.1), with an average nucleotide identity (ANI) of ≥ 95%. Genome annotation was performed using Prokka v1.13 (Seemann, 2014). Multilocus sequence typing (MLST), virulence gene profiles (including *iuc*, *rmpA*/*rmpA2*, *ybt*, *iro*, and *clb* gene clusters), and acquired antimicrobial resistance genes were identified using Kleborate v2.0.1 (Lam et al., 2021). Additional virulence gene annotation was performed using Abricate v1.0.1^2^ with the VFDB database (Zhou et al., 2025). Antimicrobial resistance genes were further annotated using AMRFinderPlus v3.11.8 (Feldgarden et al., 2021) against the NCBI Reference Gene Catalog. Plasmid sequences were identified and classified using MOB-suite v3.1.9 (Robertson and Nash, 2018), specifically the mob-recon component for contig classification. Plasmid replicon types were determined using PlasmidFinder v2.1.6 (Carattoli and Hasman, 2020).

### Phylogenomics and genomic epidemiology

For the 102 Qujing isolates, a core-genome phylogeny was reconstructed from single-copy orthologous proteins identified with OrthoFinder v3.1.0 (Emms et al., 2026). Protein sequences of 3,350 single-copy core genes were aligned using MAFFT v7.505 (Nakamura et al., 2018), and poorly aligned regions were removed using TrimAl v1.4.rev15 (Capella-Gutiérrez et al., 2009). A maximum likelihood phylogeny was inferred using IQ-TREE v3.0.1 (Wong et al., 2026) with 1,000 bootstrap replicates. The tree was visualized using iTOL (Letunic and Bork, 2024). To achieve fine-scale resolution within the dominant ST11 lineage among these isolates, a single-nucleotide polymorphism (SNP)-based phylogenetic analysis was performed. SNPs were identified using Snippy v4.6.0^3^ through reference-based mapping. A core genome SNP alignment was generated using Snippy core, and pairwise SNP distances were calculated using SNP dists v0.8.2. Putative transmission clusters were defined using a threshold of ≤15 core-genome SNPs, consistent with previously reported criteria for epidemiological linkage.

### Construction of the global *K. pneumoniae* genome dataset

Publicly available *K. pneumoniae* genomes that are completely assembled were retrieved from the NCBI Genome database using the in-house toolkit KpDBKit^4^ as of September 30, 2025. Genomes were filtered for assembly quality using CheckM v1.1.3 (completeness ≥95%, contamination ≤5%) and confirmed as *K. pneumoniae* using FastANI v1.34 (ANI ≥ 95% against the type strain genome, GCF_022869665.1). Metadata, including collection date and geographic origin, were retrieved for all qualifying genomes. To reduce redundancy and minimize bias from the overrepresentation of prevalent clones, a two-step dereplication strategy was employed. First, Mash v2.3 was used to estimate genome-wide distances using MinHash sketches (Ondov et al., 2016). Within each sequence type (ST), a range of Mash distance thresholds from 0.0002 to 0.0018 (in increments of 0.0002) was evaluated. As expected, increasing the distance threshold led to a progressive reduction in the number of representative genomes. A Mash distance of 0.0012 was adopted based on a trade-off between computational feasibility for the subsequent pangenomic analysis and the retention of sufficient genomic diversity to represent population-level heterogeneity in *K. pneumoniae*. This threshold yielded a non-redundant set of 1,778 representative genomes, which was then combined with the 102 Qujing isolates to form a final dataset of 1,880 genomes for subsequent analyses.

### Pangenomics and core gene synteny network analysis

Orthologous gene clusters (OGs) were identified using OrthoFinder v3.1.0 across the 1,880 genomes. Based on the frequency of each OG across genomes, the pangenome was partitioned into four components: core (≥99% of genomes), soft core (95–99%), shell (15–95%), and cloud (<15%). Functional annotation of OGs was performed by mapping them to KEGG pathways using KEGG Mapper (Kanehisa et al., 2021). To characterize the structural organization of the core genome, we constructed a core gene synteny network based on conservation of gene adjacency across genomes. Core orthologous groups (OGs) were represented as nodes in the network. For each genome, chromosomal gene order was systematically parsed, and pairs of core OGs that were directly adjacent (i.e., without intervening genes) were identified. For each pair of adjacent core OG i and OG j , an edge was established, and its weight $W_{\mathrm{ij}}$ was defined as the proportion of genomes in which this adjacency was preserved: $W_{\mathrm{ij}}=n_{\mathrm{ij}}/N$ , where $n_{\mathrm{ij}}$ denotes the number of genomes in which OG i and OG j are immediately adjacent, and *N* is the total number of genomes analyzed. To focus on highly conserved structural relationships, only edges that $W_{\mathrm{ij}}\geq 0.9$ were retained corresponded to adjacency conserved in at least 90% of the 1,880 genomes. This stringent threshold enabled the identification of robust and evolutionarily stable gene linkages. The resulting network was visualized using Gephi v0.10.1 (Bastian et al., 2009) with the Fruchterman-Reingold layout algorithm. Within this network, core structural modules were operationally defined as connected components comprising at least three nodes (core OGs) representing conserved gene blocks maintained across most strains.

### Sequence similarity network construction for chromosomes, plasmids, and prophages

To comprehensively capture the diversity and dynamic network relationships of mobile genetic elements without losing variants present in closely related isolates, chromosome, plasmid, and prophage sequences were extracted from the complete dataset of all 4,364 genomes (4,262 public genomes and 102 Qujing isolates), rather than being restricted to the dereplicated dataset. Plasmid sequences were identified from the same genomes using MOB-suite. Prophage regions were identified using PHASTEST (Wishart et al., 2023), which classifies prophages as intact, questionable, or incomplete based on sequence similarity to known phage proteins and gene content. All identified prophage sequences were extracted for downstream analysis. Pairwise average nucleotide identity (ANI) and alignment fraction (AF) were calculated for all chromosome, plasmid, and prophage sequences using FastANI v1.34. AF was defined as the proportion of aligned sequence length to the query sequence length.

To reduce redundancy and improve network interpretability, sequences were clustered using an iterative greedy approach based on similarity thresholds tailored to each genomic element. Specifically, chromosomes and prophage sequences were clustered at ANI ≥ 99.5% and AF ≥ 95%, whereas plasmid sequences were clustered at ANI ≥ 95% and AF ≥ 95%, reflecting their higher structural diversity and recombination frequency. Within each cluster, the longest sequence was selected as the representative node, while sequences not assigned to any cluster were retained as individual nodes. For each genomic component, a sequence similarity network was constructed in which nodes represent representative sequences, and edges connect pairs of nodes that exceed predefined ANI thresholds. Thresholds were selected to balance resolution and biological interpretability, with stricter cutoffs applied to chromosomes (ANI ≥ 99.5%) to capture clonal structure, and more permissive thresholds for plasmids (ANI ≥ 95%) and prophages (ANI ≥ 90%) to reflect their greater evolutionary plasticity. Community detection was performed using the Leiden algorithm, which optimizes modularity and improves partition stability (Traag et al., 2019). Communities were identified separately for each genomic component, representing groups of sequences with high genetic similarity. Chromosomal, plasmid, and prophage communities were designated as ChrC, PC, and PAC, respectively.

### Phylogenetic analysis

Gene-level phylogenetic analyses were conducted on key plasmid-associated and chromosomally encoded loci. Plasmid-associated elements included the aerobactin loci (*iucABCD*-*iutA*) and major carbapenemase genes (*bla*_KPC_, *bla*_NDM_, *bla*_OXA_). Chromosomally encoded loci included β-lactamase genes (*bla*_SHV_, *bla*_TEM_), the fosfomycin resistance gene *fosA*, and virulence-associated genes (*entAB*, *fepC*, *ompA*, *yagV*-*yagK* cluster). Gene sequences were extracted from genome assemblies and aligned using MAFFT v7.505, and maximum-likelihood phylogenetic trees were constructed using IQ-TREE v3.0.1 with 1,000 bootstrap replicates. Lineage assignment of the *iuc* and *ybt* loci was performed using BLASTn v2.12.0 (Altschul, 1997) against curated reference databases in BIGSdb (Jolley and Maiden, 2010).

### Statistical analysis

Statistical analyses were performed using R v4.2.0. Comparisons of continuous variables across groups were assessed using the Kruskal-Wallis test, followed by Dunn’s *post hoc* test. Categorical variable associations were evaluated using Fisher’s exact test or chi-square test as appropriate. Pearson and Spearman correlation coefficients were calculated to assess relationships between community counts and ANI thresholds. A *p*-value < 0.05 was considered statistically significant.

## Results

### Population structure and nosocomial transmission of *K. pneumoniae* in Qujing cohort

The cohort from Qujing Central Hospital from August 2024 to December 2025 was predominantly male (84.3%) and elderly (≥50 years, 69.5%). Respiratory specimens, particularly sputum, were the most common source (Supplementary Figure S1). Phylogenomic analysis of 3,350 single-copy orthologous proteins revealed substantial genetic diversity, with isolates distributed among 45 sequence types (STs) (Figure 1A). Within this diverse population, ST11 was the dominant lineage (24.5%), and all ST11 isolates harbored *bla*_KPC-2_, consistent with a carbapenem-resistant phenotype. Notably, classical hypervirulent lineages, including ST23 and ST86, were also detected, indicating the co-circulation of multidrug-resistant and hypervirulent clones within the regional population. Virulence profiling showed that yersiniabactin was the most prevalent siderophore (70.6%), followed by RmpADC (63.7%) and salmochelin (48.0%). The aerobactin loci (*iucABCD*-*iutA*) were identified in 53 isolates. All 53 isolates met the Kleborate criteria for hypervirulent *K. pneumoniae* (HvKp). Among these, 12 isolates were classified as hypervirulent carbapenem-resistant *K. pneumoniae* (Hv-CRKp), all of which belonged to ST11; the remaining 41 isolates were carbapenem-susceptible HvKp (Supplementary Table S1).

**Figure 1:**
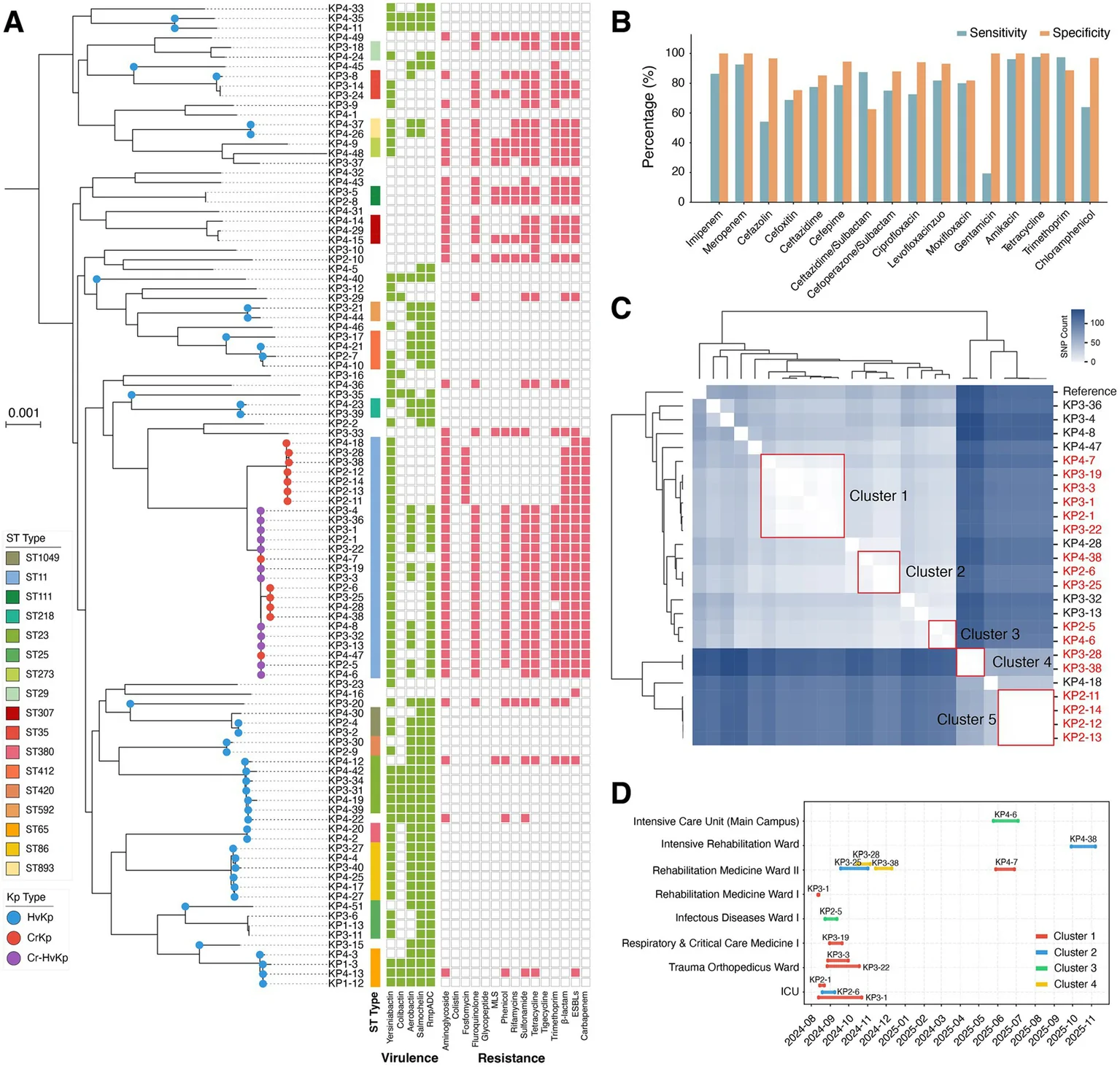
Population structure, resistance and virulence profiles, and nosocomial transmission of *Klebsiella pneumoniae* isolates from Qujing. **(A)** Phylogenomic tree based on 3,350 single-copy core genes, with branches labeled by phenotype: hypervirulent (Hv, carrying *iucABCD*-*iutA*), carbapenem-resistant (CR, carrying *bla*_KPC-2_), and hypervirulent-carbapenem-resistant (Hv-CRKp, carrying both). **(B)** Concordance between genotypic resistance determinants and phenotypic susceptibility. **(C)** Pairwise core genome SNP distance matrix for 25 ST11 isolates; clusters (≤15 SNPs) are outlined in red and labeled (Clusters 1–5). **(D)** Temporal and spatial distribution of ST11 transmission clusters by collection date and hospital ward.

To assess the utility of whole-genome sequencing (WGS) for predicting antimicrobial resistance, genotypic predictions were compared with phenotypic susceptibility testing (Figure 1B). High concordance (sensitivity and specificity both > 85%) was observed for carbapenems, tetracyclines, and trimethoprim, indicating that resistance to these drug classes is largely driven by well-characterized acquired resistance determinants. In contrast, notable discrepancies were observed with specific antibiotics. Gentamicin showed markedly low sensitivity (19.4%), despite perfect specificity, indicating that a substantial proportion of phenotypically resistant isolates lacked corresponding resistance determinants in current databases. Similarly, cefazolin showed reduced sensitivity, suggesting that resistance may be mediated by mechanisms not fully captured by gene-based prediction, such as regulatory or structural changes that affect drug permeability or expression. Cefepime and fluoroquinolones (e.g., levofloxacin) showed intermediate concordance, reflecting partial coverage of their resistance mechanisms by current genomic methods (Supplementary Table S2).

To investigate potential nosocomial transmission events, high-resolution SNP analysis was performed on 25 ST11 isolates, which represent the dominant carbapenem-resistant lineage in this cohort. Pairwise core-genome SNP distances were computed using a reference-based approach, and transmission clusters were defined using a stricter ≤ 15 SNP threshold, consistent with a previously established threshold for potential transmission (Zhang et al., 2022). Finally, we identified five transmission clusters (Figure 1C). Among these, Cluster 5 comprised four isolates recovered from different specimens from the same patient (blood, sputum, urine, and feces). However, the remaining four clusters (Clusters 1–4), comprising 13 isolates, provided strong genomic evidence of inter-patient transmission across multiple hospital units. Cluster 1, the largest group, included six isolates collected over a 10-month period (August 2024 to June 2025). Integration of genomic and epidemiological data revealed overlapping ICU, Traumatic Orthopedics, and Rehabilitation Medicine hospitalizations, with some pairs showing direct spatiotemporal links within the same wards (Figure 1D). Similarly, Cluster 4 indicated localized transmission within the Rehabilitation Medicine Ward II, with two isolates (KP3-28 and KP3-38) collected within a one-month interval (October 15, to November 14, 2024), and partially overlapping hospitalization periods (Oct 15–Nov 5 and Nov 14–Dec 10, respectively), supporting short-term within-ward transmission. In contrast, Clusters 2 and 3 exhibited more complex dynamics: Cluster 2 included isolates with limited spatial overlap but temporal proximity, suggesting indirect transmission via shared environments, whereas isolates in Cluster 3 were separated by a year, indicating either the long-term persistence of a specific clone or repeated introductions from a common source. In summary, high-resolution genomic analysis identified four distinct ST11 transmission clusters exhibiting both direct spatiotemporal linkages and potential environmental transmission dynamics in this regional cohort.

### Global pangenome architecture and core genome structural conservation

To place the Qujing isolates in a global evolutionary context, we compiled a dataset of publicly available complete *K. pneumoniae* genomes. A total of 4,262 genomes with curated metadata (geographic origin and collection time) were retrieved (Supplementary Figure S2; Supplementary Table S3). These genomes spanned six continents and more than 40 countries, though sampling was uneven, with China, the United States, and Norway accounting for the majority of samples. To reduce bias from overrepresented epidemic clones, we applied a dereplication strategy using Mash distance within each sequence type (ST). Within each ST, genomes with a pairwise Mash distance <0.0012 were grouped, and a single representative genome was selected from each group, yielding a non-redundant dataset of 1,778 genomes. This dataset was then combined with the 102 Qujing isolates to create a final dataset of 1,880 genomes. Within this non-redundant global dataset, 135 isolates (7.18%) were identified as Hv-CRKp (defined by carbapenemase genes and a Kleborate virulence score ≥3), with the ST11 lineage predominated (28.89%, 39/135). Protein sequence clustering identified 16,073 orthologous groups (OGs), which were then classified into core (3952), soft core (288), shell (1360), and cloud (10473) OGs based on population frequency (Supplementary Figure S3A). Consistent with previous reports, the *K. pneumoniae* pangenome exhibited an open structure with a large cloud reservoir. Nevertheless, core genes accounted for nearly 80% of total gene occurrences across genomes (Supplementary Figure S3B), a proportion higher than commonly recognized. Functional annotation confirmed that core genes are enriched in essential metabolic pathways, whereas accessory genes are linked to environmental adaptation and host interaction (Supplementary Figure S3C).

To this end, we constructed a core gene synteny network with the 3,952 core OGs as nodes, with edges defined by strict chromosomal adjacency and weighted by genome-wide conservation (Figure 2A). The resulting network comprised 3,952 nodes (core OGs), with edges retained only when the pairwise adjacency was conserved in ≥90% of the 1,880 genomes, thereby highlighting only the most robust syntenic relationships. Of these, 3,673 nodes (92.94%) were non-isolated, each connected by at least one edge conserved in ≥90% of the 1,880 genomes. This indicates that the vast majority of core genes are organized into structurally coherent genomic blocks. The interconnected nodes were further grouped into syntenic modules of varying sizes, ranging from small chains of 3–5 genes to a large module containing nearly 60 genes. The module size distribution showed a clear decay pattern: the number of modules decreased rapidly as module size increased (Figure 2B). This distribution indicates that although most core gene adjacencies are confined to small, tight clusters, a subset of core genes has been preserved as large, multi-gene syntenic blocks across the vast majority of *K. pneumoniae* genomes. The analyses above focused exclusively on edges with a conservation frequency ≥0.9, which represent the most robust syntenic relationships. However, examination of the full edge weight distribution revealed a striking bimodal pattern (Figure 2C). A large number of edges were either conserved in very few genomes (weight ≤0.02) or in nearly all genomes (weight ≥0.9), with very few edges falling in the intermediate range (0.1–0.8). This distribution suggests that core gene adjacencies are generally either highly labile (present in only a small subset of genomes) or highly conserved (present in ≥90% of genomes), with few intermediate states. Thus, while the core genome of *K. pneumoniae* is not randomly organized, its synteny is markedly non-conserved: most conserved adjacencies involve only a few genes, and only a small fraction of the core genome is maintained as large, multi-gene structural blocks. Importantly, these conserved modules harbored key resistance genes (*fosA*, *bla*_SHV_), virulence determinants (enterobactin synthesis genes *entAB* and the ECP fimbrial cluster *yagVWXYZK*), and prophage-associated genes, indicating that functionally important traits are embedded in the stable chromosomal backbone.

**Figure 2:**
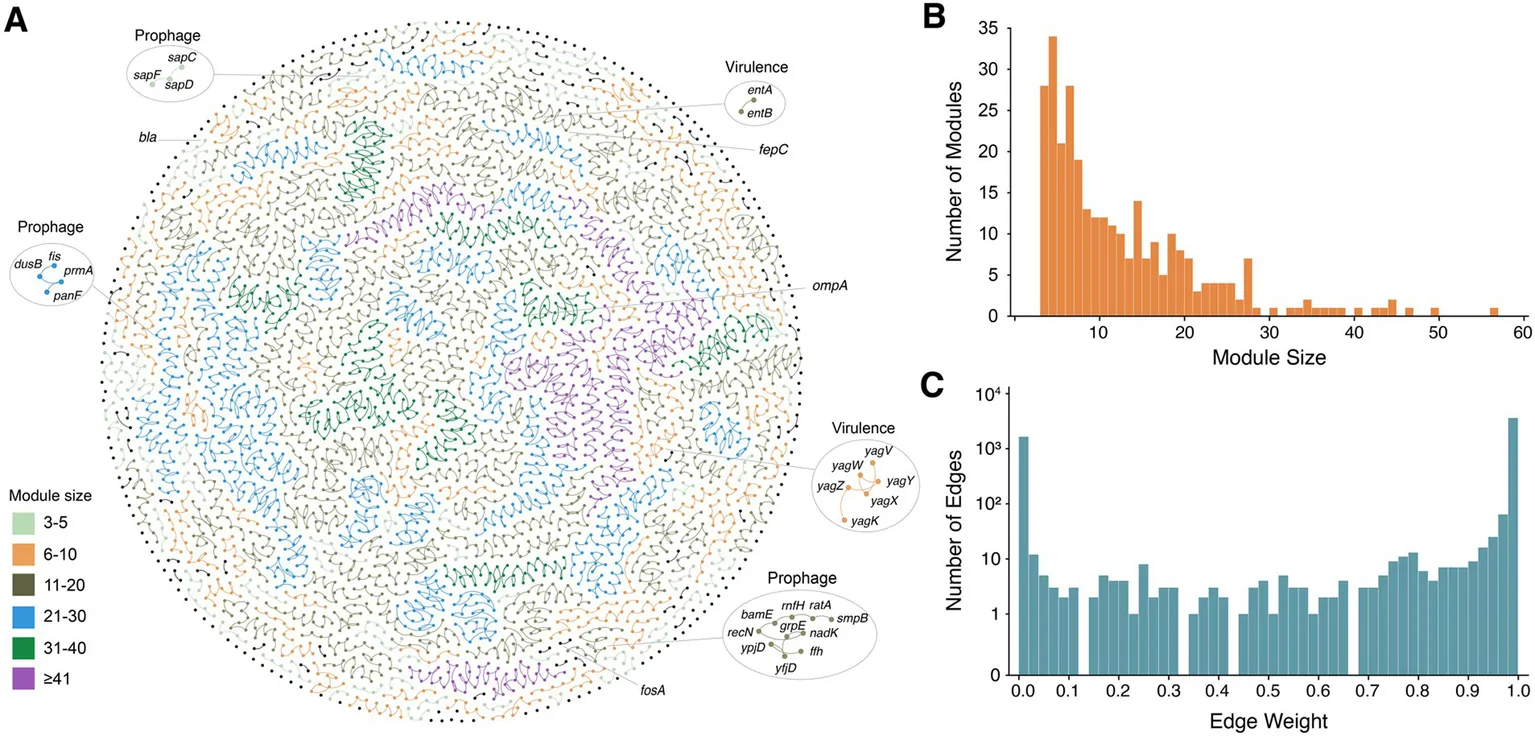
Conserved synteny modules in the *K. pneumoniae* core genome. **(A)** Synteny network constructed using high-confidence edges (weight ≥0.9), with nodes representing orthologous groups and edges indicating conserved adjacency; modules (conserved gene blocks) are identified by community detection. **(B)** Size distribution of core structural modules, highlighting the predominance of small modules and the presence of larger blocks containing virulence- and prophage-associated genes. **(C)** Distribution of edge weights in the core gene synteny network; edge weight reflects the frequency of strict gene adjacency across genomes.

### Plasmid population structure and its association with the chromosome

While the chromosomal core genome provides a stable structural backbone, the accessory genome—especially plasmids—drives rapid adaptation through horizontal gene transfer. To quantitatively assess this contrast, we extracted 9,240 plasmid sequences from 4,364 genomes and computed pairwise average nucleotide identity (ANI) and alignment fraction (AF) for both chromosomes and plasmids. As expected, chromosomes were highly conserved: most pairwise comparisons showed ANI > 99% and AF consistently above 95% (Supplementary Figure S4). In contrast, plasmids exhibited substantially greater diversity. Plasmid ANI values were broadly distributed between 95 and 100% (Supplementary Figure S5A), and plasmid AF values spanned a wide range from 50 to 100% with a near-uniform distribution (Supplementary Figure S5B). Thus, plasmid pairwise similarity exhibited a broader distribution than chromosomal similarity across the analyzed dataset.

To investigate whether associations exist between chromosomal background and plasmid diversity, we constructed a chromosome similarity network in which nodes represented individual chromosomes and edges were weighted by pairwise ANI values. To define discrete chromosomal clusters, we examined community counts across a range of ANI thresholds. The number of communities increased sharply before plateauing at ≥99.5% (Supplementary Figure S6), supporting the use of this threshold to define chromosomal communities (ChrCs). At ANI ≥ 99.5%, the chromosome network was partitioned into 345 communities (Supplementary Table S4), showing a high degree of concordance with MLST-defined lineages. The majority of sequence types (STs) were confined to single chromosomal communities; however, a small number of STs (10 out of 631) were distributed across multiple communities (Supplementary Table S5). Given the substantially larger number of plasmid sequences, we performed dereplication using stringent criteria (ANI ≥ 99.5% and AF ≥ 95%), yielding 2,530 representative plasmid nodes. Then, a plasmid similarity network was constructed with a lower edge threshold of ANI ≥ 95%, reflecting the greater sequence diversity among plasmids. Community detection using the Leiden algorithm identified 136 plasmid communities (PCs) (Figure 3; Supplementary Table S6). Notably, plasmid community sizes were highly uneven: two dominant communities, PC4 and PC0, together accounted for about half of all plasmid representatives, whereas most communities were small and lineage-restricted.

**Figure 3:**
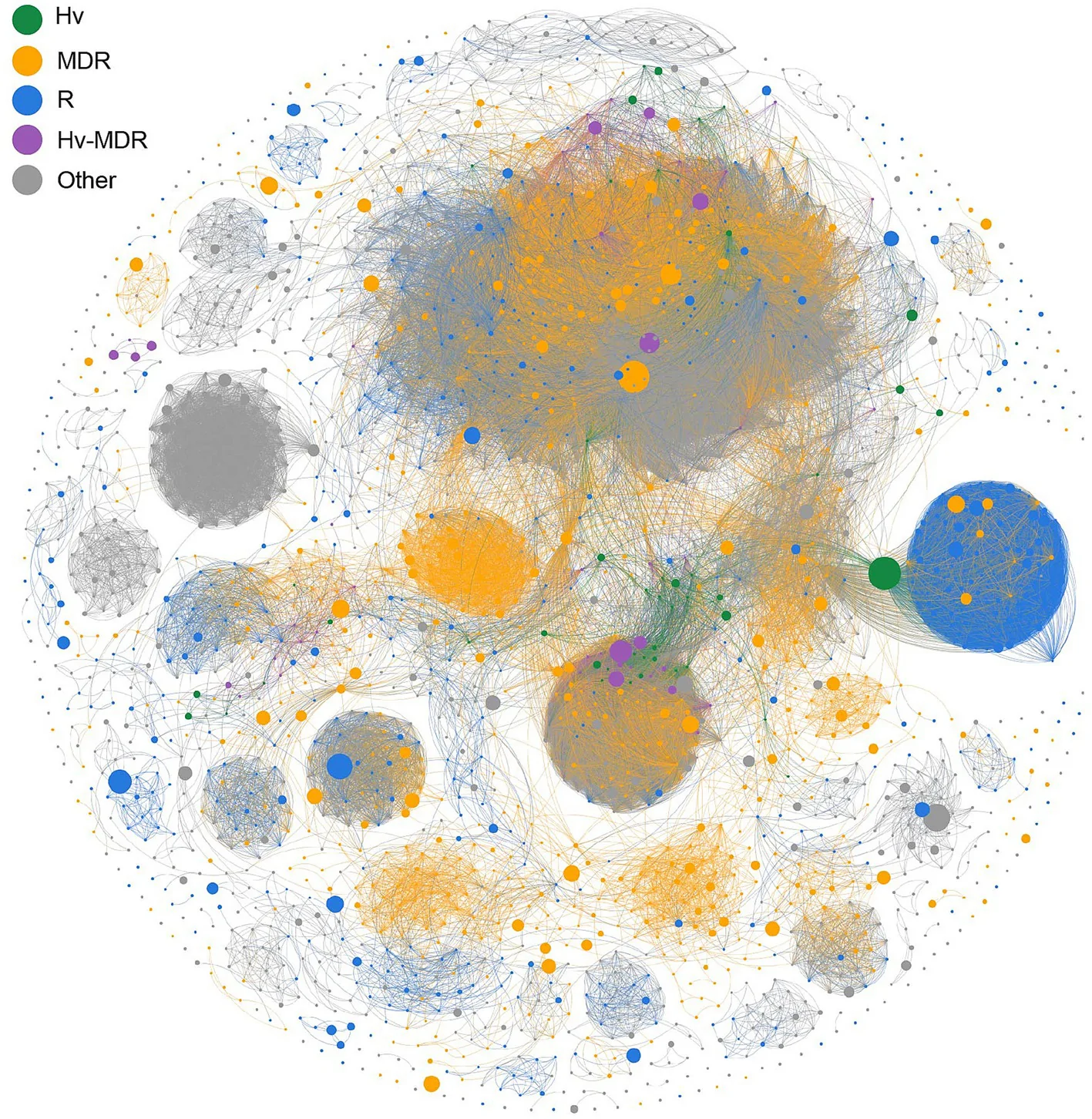
Plasmid similarity network of *K. pneumoniae*. Nodes represent representative plasmid sequences (dereplicated at ANI ≥ 99.5% and AF ≥ 95%). Edges connect pairs with ANI ≥ 95%. Node colors indicate phenotypic categories.

Plasmid communities differed markedly in their structural and functional characteristics. Plasmid size varied significantly across PCs (Kruskal-Wallis test, *p* < 0.0001), and PC5 harbored the largest plasmids (median ~272 kb) (Supplementary Figure S7). Functional classification revealed strong specialization: PC4 was enriched for hypervirulence (Hv) and multidrug resistance (MDR) plasmids, whereas PC0 was dominated by simpler resistance (R) plasmids; other communities showed mixed or niche-specific compositions. Further analysis of plasmid functional types revealed strong associations with replicon types, host lineages, and geographic origin (Supplementary Figure S8). Hypervirulence plasmids were predominantly associated with IncHI1B and IncFIB replicons and were enriched in classical hypervirulent lineages (ST23 and ST65). Multidrug resistance plasmids were primarily linked to ‘IncFIB; IncFII’, IncFII, and IncC replicons, whereas simpler resistance plasmids were associated with ‘IncFII; IncR’, IncL, IncFIB, and IncX3. Carbapenemase-carrying plasmids showed structured distributions (Supplementary Figure S9): ‘IncFII; IncR’ was the primary carrier of *bla*_KPC_ genes, whereas IncL was strongly associated with *bla*_OXA-48_. ST11 was the dominant host of carbapenemase plasmids, particularly those carrying *bla*_KPC-2_, which were largely carried by PC4 plasmids, highlighting the convergence of resistance and virulence within this major plasmid reservoir.

To investigate chromosome–plasmid relationships, we integrated ChrC and PC assignments into a bipartite association network, visualized as a Sankey diagram (Supplementary Figure S10). The largest chromosomal community, ChrC23 (dominated by ST11), showed extensive connectivity across multiple PCs, particularly PC4 and PC0, indicating a high capacity for plasmid acquisition and maintenance. Notably, PC4 showed a strong preferential association with ChrC23, whereas PC0 was widely distributed across multiple host lineages (e.g., ChrC25/ST307 and ChrC31/ST15). Other ChrCs displayed more restricted plasmid associations. To further elucidate the genetic background of genomes harboring multiple functional plasmid types (Hv, MDR, and Hv-MDR), we examined associations across four hierarchical levels, including chromosomal communities, plasmid communities, replicon types, and host sequence types (STs) (Figure 4A). ChrC23, dominated by ST11, accounted for the largest proportion of such genomes and exhibited the broadest plasmid community spectrum, with most plasmid flows directed toward PC4, along with additional contributions from PC5 and PC0. In contrast, other chromosomal communities exhibited more restricted associations, linking them to a limited number of plasmid communities. At the plasmid level, PC4 was the predominant community among multi-trait plasmid carriers and was primarily associated with IncHI1B and IncFII replicons. These replicon types showed clear functional differentiation, with IncHI1B enriched for hypervirulence plasmids and IncFII predominantly associated with multidrug-resistant plasmids. At the host lineage level, ST11 was the dominant genotype and was concurrently associated with IncHI1B and IncFII replicons.

**Figure 4:**
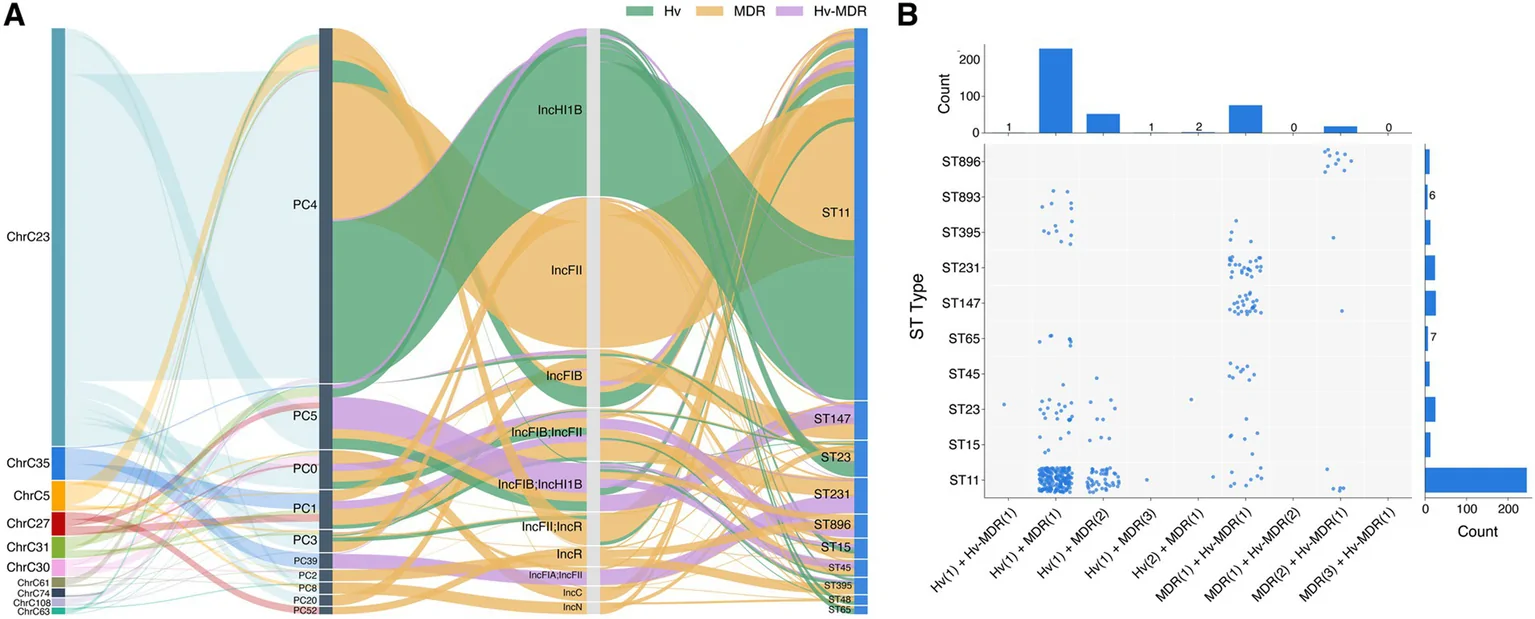
Co-carriage of distinct virulence and resistance plasmid types in *K. pneumoniae* genomes. **(A)** Sankey diagram showing the flow of genomes carrying different plasmid types (Hv: hypervirulence; MDR: multidrug resistance; Hv-MDR: hypervirulence and multidrug resistance) across chromosomal communities (ChrC), plasmid communities (PC), and sequence types (ST). Bandwidth is proportional to the number of genomes. **(B)** Distribution of co-carriage combinations of distinct plasmid types across sequence types.

Building on these association patterns, we next quantified the combinatorial configurations of functional plasmid types in genomes with multiple plasmids. Among 381 genomes harboring at least two distinct plasmid types (Hv, MDR, R, or Hv-MDR), the most common combination was Hv(1) + MDR(1) (60.6%, 231/381), and ST11 accounted for 81.0% (187/231) of these cases (Figure 4B). The second most frequent combination was MDR(1) + Hv-MDR(1) (19.9%, 76/381), primarily associated with ST147 and ST231. Beyond combinatorial patterns, we next asked whether the evolutionary modes of these traits vary by genetic location. Phylogenetic analysis further distinguished the evolutionary modes of plasmid-borne and chromosomally encoded traits (Supplementary Figures S11, S12). Plasmid-borne *iucABCD*-*iutA* exhibited a simple, clonal phylogeny dominated by a single lineage (*iuc1*, 82.4%) that was tightly associated with ST11 and PC4, consistent with horizontal spread and clonal expansion. In contrast, chromosomally encoded genes, including *entAB*, *fepC*, and *yagVWXYZK,* exhibited complex phylogenies that mirrored those of the core genome, indicating long-term vertical inheritance.

### Prophage diversity, structural conservation, and their distinct contribution to resistance gene dissemination

Prophages, which are stably integrated into bacterial chromosomes, can carry resistance genes and interact with plasmid communities. To complement the plasmid-focused analysis, we characterized the prophage population across 4,364 genomes. A total of 22,088 prophage regions were identified, averaging ~5 prophages per genome (Supplementary Table S7). Length distribution varied by genomic context (Supplementary Figure S13): intact chromosomal prophages had a larger median length (47.1 kb, IQR, 40.2–55.9 kb) than plasmid-associated prophages (39.2 kb, IQR, 32.8–45.6 kb), whereas incomplete elements were shorter (median 22.3 kb). Compared with plasmids, prophages showed markedly higher sequence conservation. Pairwise comparisons showed that 76.6% of prophage alignments had ANI > 99%, versus 22.7% for plasmids (Supplementary Figure S14A). Similarly, 31.2% of prophage comparisons had AF ≥ 90%, compared with only 3.2% for plasmids (Supplementary Figure S14B). Despite this high conservation, the large number of distinct prophage regions indicates that prophages are a structurally diverse yet sequence-constrained component of the accessory genome. Graph clustering grouped prophages into discrete communities (Figure 5A). We identified 362 prophage communities (PACs) with highly uneven sizes. The largest communities were dominated by intact chromosomal prophages (>99% of members), whereas smaller communities frequently contained mixed elements, including plasmid-associated and incomplete prophages. Additional clusters were enriched for questionable or degraded prophages, indicating different evolutionary states.

**Figure 5:**
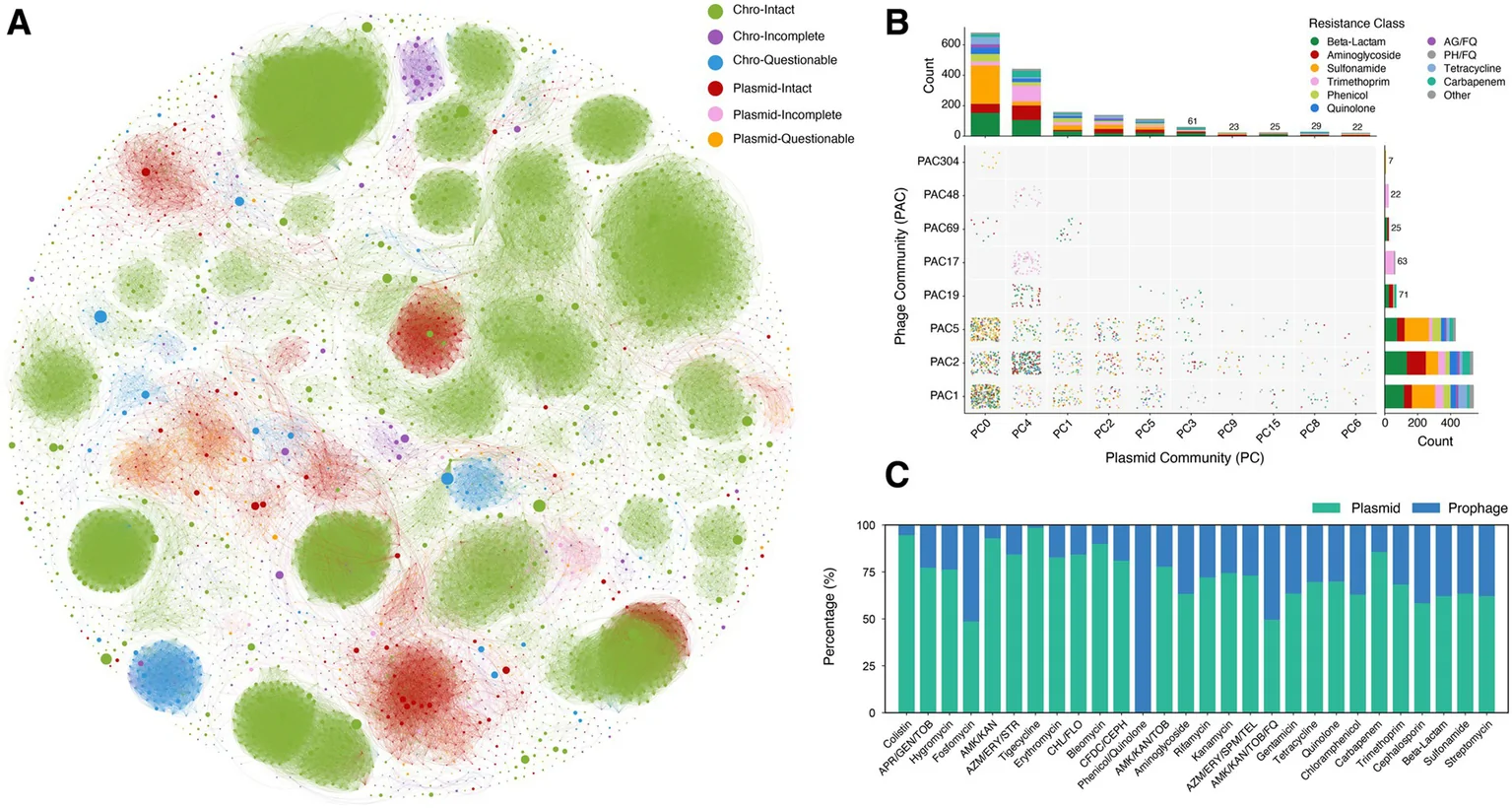
Prophage diversity, association with plasmids, and contribution to antimicrobial resistance genes. **(A)** Prophage similarity network. Nodes represent representative prophage sequences (dereplicated at ANI ≥ 99.5% and AF ≥ 95%). Edges connect pairs with ANI ≥ 90%. **(B)** Association between prophage communities and plasmid communities. Each dot represents a prophage, plotted by its assigned plasmid community (x-axis) and prophage community (y-axis). **(C)** Distribution of antimicrobial resistance genes across 28 antibiotic subclasses. Bars show the proportion of genes contributed by plasmids (green) versus prophages (blue) within each subclass.

A subset of prophages harbored antimicrobial resistance genes, accounting for 5.6% (1,242/22,020) of all prophage regions. These resistance-carrying prophages showed strong lineage and geographic biases (Supplementary Figure S15): ST11 harbored the greatest diversity and abundance, and China and the United States contributed the most diverse profiles. Resistance genes were concentrated in a small number of clusters, suggesting structured rather than random integration. Clear associations were observed between prophage and plasmid communities (Figure 5B). Major resistance-associated prophage clusters were preferentially associated with specific plasmid backbones, including multidrug-resistant and hypervirulence-associated plasmids. This non-random coupling indicates that prophages and plasmids are jointly structured within shared host backgrounds.

To dissect the roles of plasmids versus prophages in the dissemination of resistance, we analyzed AMR gene distributions across 28 antibiotic subclasses (each with >100 genes) (Figure 5C). Of 39,295 AMR annotations, 66.2% (26,008) were plasmid-derived, and 33.8% (13,287) were associated with prophages carried on plasmids. Plasmids dominated the dissemination of resistance genes, accounting for >50% of genes in 21 of 28 categories. This dominance was pronounced among critical antibiotics: carbapenems (85.6%, *n* = 2,284), colistin (94.6%, *n* = 111), and tigecycline (98.6%, *n* = 277). Other subclasses with strong plasmid bias (>80%) included aminoglycosides (amikacin/kanamycin, 92.9%), bleomycin (89.9%), chloramphenicol/florfenicol (84.2%), and macrolide-related groups (84.2%). Even in high abundance categories such as streptomycin (*n* = 5,386), sulfonamides (*n* = 4,485), and β-lactams (*n* = 3,753), plasmid contributions remained stable at ~62–63%. By contrast, prophage-associated resistance genes showed distinct, non-random enrichment patterns. Phenicol/quinolone resistance was entirely prophage-associated (100%, n = 595), and no plasmid-borne homologs were detected. Fosfomycin resistance was also associated with prophage predominance (51.3%, *n* = 191). A subset of subclasses showed balanced contributions (40–60%). For example, the combined aminoglycoside/quinolone/tobramycin category showed nearly equal contributions (49.5% plasmid vs. 50.5% prophage), suggesting the coexistence of multiple horizontal transfer mechanisms within the same resistance gene pools. Together, these results establish a multi-layer genomic framework: a stable core genome organized into conserved synteny modules, a structured plasmid ecosystem with functional specialization (PC4/PC0), and a distinct but interconnected prophage network.

### Plasmid population in the Qujing cohort

We further examined the plasmid community structure among the Qujing isolates, which largely mirrored global patterns (Supplementary Figure S16). PC4 was also the largest plasmid community in Qujing (90 plasmids) and exhibited the broadest ST distribution, with the strongest association with ST11. Resistance gene profiles were similar to those in the global dataset: PC4 harbored diverse resistance genes (β-lactams, aminoglycosides, quinolones), while PC0, PC3, and PC6 harbored distinct combinations of resistance genes. Virulence genes were mainly distributed in PC4, which carried the majority of aerobactin, *rmpADC*, and salmochelin genes, while PC6 served as the primary reservoir of yersiniabactin and was the only community to carry both aerobactin and yersiniabactin. Plasmid size varied across communities, with PC5 and PC6 harboring larger plasmids than PC4.

Among the Qujing isolates, we identified a highly unusual PC4 plasmid, KP4-12-2, recovered from an ST23 strain. This plasmid (~350 kb) belongs to PC4 and carries an extraordinary combination of genetic features (Figure 6A). First, it harbors two complete, distinct copies of the *iucABCD*-*iutA* clusters and two copies of the *iroBCDN* salmochelin cluster – a configuration never reported in *K. pneumoniae*. Pairwise comparisons between the two copies revealed high nucleotide identity for *iutA* (99.68%), *iucB* (92.93%), and *iucC* (89.97%), but lower identity for *iucD* (78.72%) and *iucA* (76.93%) (Supplementary Table S1; Figure 6B). For the *iro* cluster, the two copies showed 89.16% identity for *iroB*, 85.77% for *iroD*, 47.52% for *iroC*, and 30.02% for *iroN*. These divergent identities suggest that the two copies originated from distinct ancestral sources and were assembled onto the same plasmid via recombination or modular exchange. Second, KP4-12-2 also harbors additional hypervirulence determinants: *peg-344* (a hypervirulence marker) and the *rmpADC* cluster (regulators of the mucoid phenotype). Third, it harbors multiple resistance genes that confer resistance to aminoglycosides, sulfonamides, tetracyclines, and chloramphenicol, making it both a hypervirulence plasmid and a multidrug-resistance plasmid (Hv-MDR). Structural analysis revealed that the two copies of the *iuc* and *iro* clusters are located in distinct regions of the plasmid, each flanked by different replicons and insertion sequences (IS3, IS110, and IS6 families), supporting a model of modular evolution through plasmid fusion or sequential integration. This plasmid is the first report of multicopy *iuc* and *iro* clusters on a single *K. pneumoniae* plasmid, providing new insight into the evolution of hypervirulence. In addition to KP4-12-2, two other Hv-MDR plasmids (KP3-8-2 and KP3-20-2) were identified in the Qujing isolates, both belonging to the PC0 community (~250 kb each; Supplementary Figure S17). In contrast to the mosaic structure of KP4-12-2, these PC0 Hv-MDR plasmids displayed a more conserved architecture. Both carried IncFIB and IncFII replicons, a single complete aerobactin gene cluster (*iucABCD*-*iutA*), and a consistent set of resistance genes conferring resistance to sulfonamides (*sul2*), aminoglycosides (aph(3″)-Ib, aph(6)-Id), tetracyclines (tet(A)), and chloramphenicol (*floR*). These contrasting architectures—multi-module fusion in PC4 versus a conserved structure in PC0—indicate that Hv-MDR plasmids have evolved along distinct trajectories.

**Figure 6:**
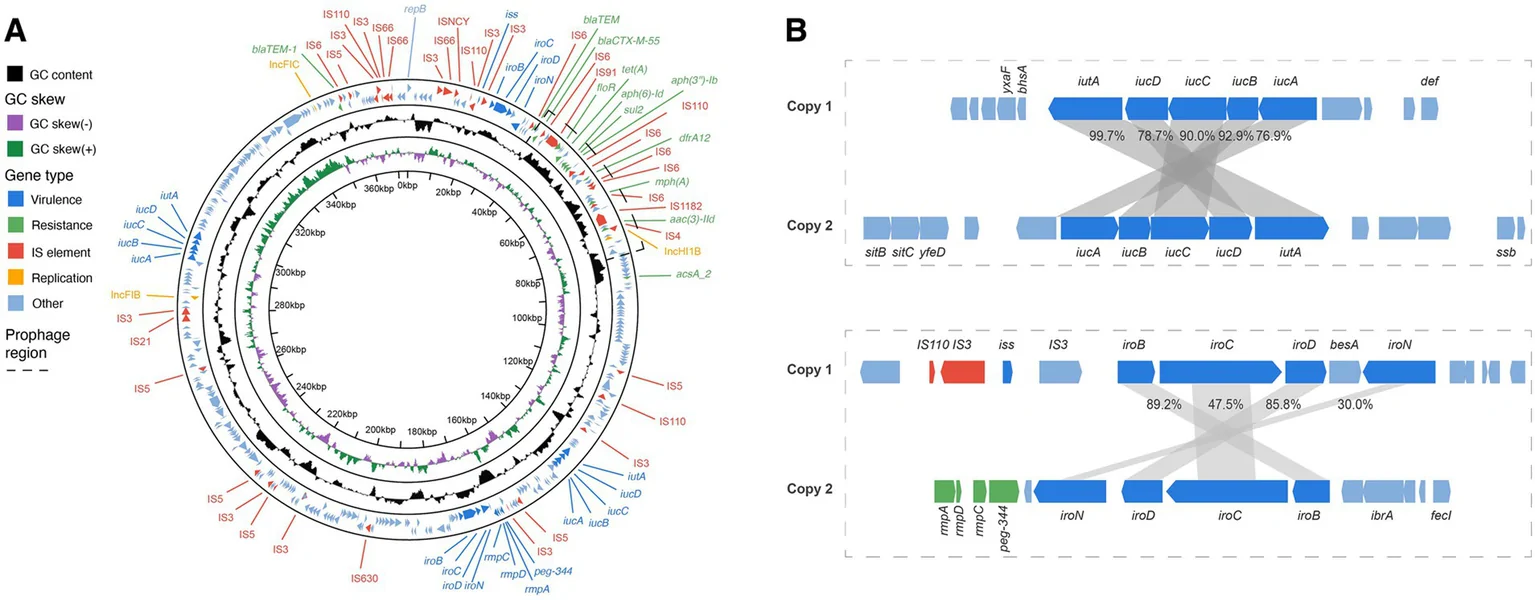
Genomic architecture of the multi-copy virulence plasmid KP4-12-2. **(A)** Circular map of plasmid KP4-12-2 (~350 kb). From inner to outer: size scale, GC skew (−), GC skew (+), GC content, and annotated genes. **(B)** Pairwise alignment of the two *iucABCD*-*iutA* copies (upper panel) and the two *iroBCDN* copies (lower panel). Colored shading between the two copies indicates sequence identity; wider shading indicates greater similarity.

## Discussion

The convergence of hypervirulence and carbapenem resistance in *Klebsiella pneumoniae* represents a major challenge for global public health. By integrating regional genomic surveillance with global population genomics, this study establishes a network-based, multi-layer framework linking chromosomal structure, plasmid ecology, and prophage dynamics (Figures 1–5). Our integrated genomic analysis demonstrates that this convergence is not random, but strongly shaped by lineage-specific plasmid compatibility within a structurally constrained chromosomal background.

The ST11 lineage has emerged as the dominant carbapenem-resistant clone in East Asia and plays a disproportionate role in the convergence of resistance and hypervirulence (Xie et al., 2021). At the global scale, ST11 exhibits the highest connectivity to plasmid communities, with a particularly strong association with the PC4 community, which is enriched for hypervirulence (Hv) and multidrug resistance (MDR) plasmids (Lan et al., 2021). Multi-trait plasmid combination analysis shows that ST11 accounts for 81.0% of strains with the Hv(1) + MDR(1) combination—the most common convergence pattern. Our quantitative plasmid combination analysis indicates that ST11 serves as a privileged host for acquiring and maintaining co-existing hypervirulence and multidrug resistance plasmids, acting as a major conduit for trait convergence. In contrast, other high-risk clones display more restricted plasmid profiles: ST258 is primarily associated with classical resistance plasmids, whereas ST307 and ST340 are largely associated with MDR plasmids and show minimal acquisition of hypervirulence elements. These observations support a lineage-specific convergence model in which chromosomal background strongly shapes the ability to acquire and maintain high-risk plasmids, thereby establishing ST11 as a primary dissemination hub.

Beyond accessory genome dynamics, the core genome of *K. pneumoniae* exhibits a highly organized structural architecture. Our findings show that core genes account for nearly 80% of total gene occurrences, underscoring the evolutionary and functional centrality of the core backbone and suggesting that the overall genomic diversity of *K. pneumoniae* may have been somewhat overestimated in earlier studies (Wyres and Holt, 2016; Wyres et al., 2020). The majority of core genes are embedded within conserved synteny modules that preserve stable genomic neighborhoods across the global population. This organization extends beyond conservation of gene content to conservation of position, indicating strong evolutionary constraints on genome structure. Notably, in addition to intrinsic resistance genes (*fosA* and *bla*_SHV_), elements traditionally considered mobile or accessory, such as *bla*_TEM_, were consistently integrated within core structural modules (Martin and Bachman, 2018). Similarly, virulence-associated loci, such as the ECP fimbrial cluster and the enterobactin-related genes (*entAB*, *fepC*), are stably incorporated. This organization shows strong evolutionary constraints on gene content and positional conservation, providing a mechanistic basis for why certain lineages preferentially accumulate complex plasmids. The modular organization likely reflects functional coupling and co-regulation, enabling horizontally acquired sequences—including prophage-derived elements—to become evolutionarily stabilized within the chromosome (Fondi et al., 2024; Brezner et al., 2025).

Plasmid population structure is highly organized into discrete communities with distinct functional profiles. The PC4 community is the primary reservoir of combined virulence and resistance determinants, whereas PC0 is dominated by classical resistance plasmids with a broader host distribution. The strong associations between plasmid communities and host lineages underscore the role of host–plasmid compatibility in shaping patterns of dissemination. PC4 plasmids are tightly linked to ST11, which serves as a major recipient and dissemination hub, whereas PC0 plasmids are distributed across multiple lineages, consistent with a broader host range. This stratified plasmid ecosystem offers a mechanistic explanation for the uneven distribution of high-risk traits: lineages capable of maintaining complex plasmids, such as ST11, are more likely to acquire both virulence and resistance determinants, thereby driving the emergence of high-risk clones.

Prophages show high sequence conservation yet retain distinct community structures. The majority of prophage sequences show near-identical nucleotide identity, consistent with dominant vertical inheritance and limited diversification relative to plasmids. Despite this conservation, prophages form discrete communities that exhibit non-random associations with plasmid communities, suggesting coordinated structuring of mobile genetic elements within shared host backgrounds. Functionally, prophages contribute less to resistance gene carriage than plasmids but show specificity for certain resistance categories. The complete or strong enrichment of specific AMR categories (such as phenicol/quinolone and fosfomycin) within prophage regions suggests that certain resistance determinants may preferentially rely on phage-mediated transduction rather than plasmid conjugation for dissemination. Integration into the chromosome and high sequence conservation indicate that prophages serve as stable reservoirs of genetic material that can be mobilized under selective pressure, thereby reinforcing the multi-layered network underlying trait convergence.

Integrating genomic surveillance with population-scale analysis provides actionable insights for infection control (Sundermann et al., 2026). The identification of ST11 transmission clusters within specific hospital departments underscores the pivotal role of ICU and surgical departments as nosocomial transmission hubs (Jiang et al., 2024), highlighting an urgent need for targeted infection control measures to curb the spread of this high-risk lineage. Incorporating core genome SNP analysis into routine surveillance could enable earlier intervention in high-risk settings. The detection of Hv-CRKp strains within the ST11 lineage further underscores the clinical significance of plasmid-mediated convergence. These strains combine resistance and virulence, limiting treatment options and increasing the risk of severe infection. Targeted molecular screening for key virulence and resistance markers may enhance early detection. At the therapeutic level, genotype–phenotype concordance varies among antibiotic classes, highlighting both the primary strengths and clear limitations of WGS-based resistance prediction in clinical settings. While genomic prediction is reliable for several drug categories, discrepancies in others indicate that phenotypic testing remains necessary. These inconsistencies likely reflect complex resistance mechanisms beyond the mere presence of resistance genes. The global distribution patterns observed here also underscore the need for region-specific surveillance strategies. The dominance of distinct clones and plasmid communities across continents suggests that intervention strategies must be tailored to local epidemiological contexts.

Several limitations should be acknowledged. First, the analysis was limited to complete genomes, potentially underrepresenting the diversity present in draft assemblies. Second, the associations between chromosomal lineages and plasmid communities are correlative and require experimental validation. Third, the lack of temporal data limits inference about transmission dynamics. Finally, the lack of clinical outcome data prevents a direct link between genomic features and patient prognosis. Future studies that integrate long-read sequencing, functional assays, and expanded global sampling will be essential to refine our understanding of lineage-specific plasmid compatibility and the mechanisms underlying the emergence of high-risk *K. pneumoniae* clones. In addition, experimental studies will be essential for elucidating the mechanistic basis of plasmid compatibility and stability. Expanding global sampling, particularly in underrepresented regions, will further refine our understanding of *K. pneumoniae* population dynamics.

In summary, this study establishes a network-based, multilayer framework that connects chromosomal structure, plasmid ecology, and prophage dynamics. Our findings reveal that lineage-specific plasmid compatibility within a structurally constrained genome drives the convergence of hypervirulence and carbapenem resistance, offering both mechanistic insight and actionable guidance for surveillance and control of high-risk *K. pneumoniae* lineages.

## Data Availability

The complete genomic data for all new isolates of *Klebsiella pneumoniae* have been deposited in the China National Genomics Data Center (NGDC) under Bioproject: PRJCA061509. The detailed accession numbers are listed in Supplementary Table S1. The curated datasets, sequence-similarity network files, and all custom scripts for figure generation have been deposited at Zenodo (DOI:10.5281/zenodo.21150875) to ensure full reproducibility of the multi-layer genomic network analyses.

## Footnotes

1
https://github.com/nanoporetech/dorado/

2
https://github.com/tseemann/abricate

3
https://github.com/tseemann/snippy

4
https://gitee.com/mselab_admin/kp-dbkit
