# Supplementary material for: A central hub for convergence: multi-layer genomic networks reveal lineage-specific plasmid compatibility driving hypervirulence and carbapenem resistance in ST11 Klebsiella pneumoniae
Source: Front Microbiol. 2026 Sep 30;17:1932987. doi: 10.3389/fmicb.2026.1932987 (PMC13661990; doi:10.3389/fmicb.2026.1932987)

Supplementary materials for

A central hub for convergence: Multi-layer genomic networks reveal lineage-specific plasmid compatibility driving hypervirulence and carbapenem resistance in ST11 *Klebsiella pneumoniae*

Xuan Hai[1,†], Jun-Ling Wang[2,†], Qi Chen[2], Yi-Fei Lu[1], Xiu-Ping Li[2], Cong-Jian Li[1], Chao-Fu Yue[2], Jian-Mei Song[2], Ling-Ling Yang[1], Min Chen[2], Hong-Wei Li[2,*], and Xiao-Yang Zhi[1,*]

[1] Yunnan Institute of Microbiology, Key Laboratory of Microbial Diversity in Southwest China of Ministry of Education, School of Life Sciences, Yunnan University, Kunming 650091, People's Republic of China.

[2] Center of Clinical Laboratory, Qujing Central Hospital, Qujing Affiliated Hospital of Kunming Medical University, Qujing 655000, People's Republic of China

† These authors contributed equally to the work.

* Authors for correspondence: Hong-Wei Li, lihongwei1104@sina.com; Xiao-Yang Zhi, xyzhi@ynu.edu.cn

Supplementary tables

All supplementary tables are organized in a Microsoft Excel file named supplementary_tables.xlsx.

Supplementary Table S1. Genomic features, resistance genes, and virulence markers of 102 K. pneumoniae isolates from Qujing.

Supplementary Table S2. Antibiotic susceptibility profiles of 102 K. pneumoniae isolates from Qujing.

Supplementary Table S3. Genomic and epidemiological metadata for K. pneumoniae reference genomes.

Supplementary Table S4. Clustering of K. pneumoniae genomes into chromosomal communities.

Supplementary Table S5. Sequence type (ST) distribution within and between chromosomal communities.

Supplementary Table S6. Community assignment, replicon typing, and phenotypic annotation of plasmid nodes.

Supplementary Table S7. Genomic features and community classification of identified prophages.

Fig. S1. Demographic and clinical characteristics of 102 *K. pneumoniae* isolates from Qujing. (A) Sex distribution. (B) Age distribution. (C) Sample source distribution, showing a predominance of respiratory specimens (e.g., sputum).

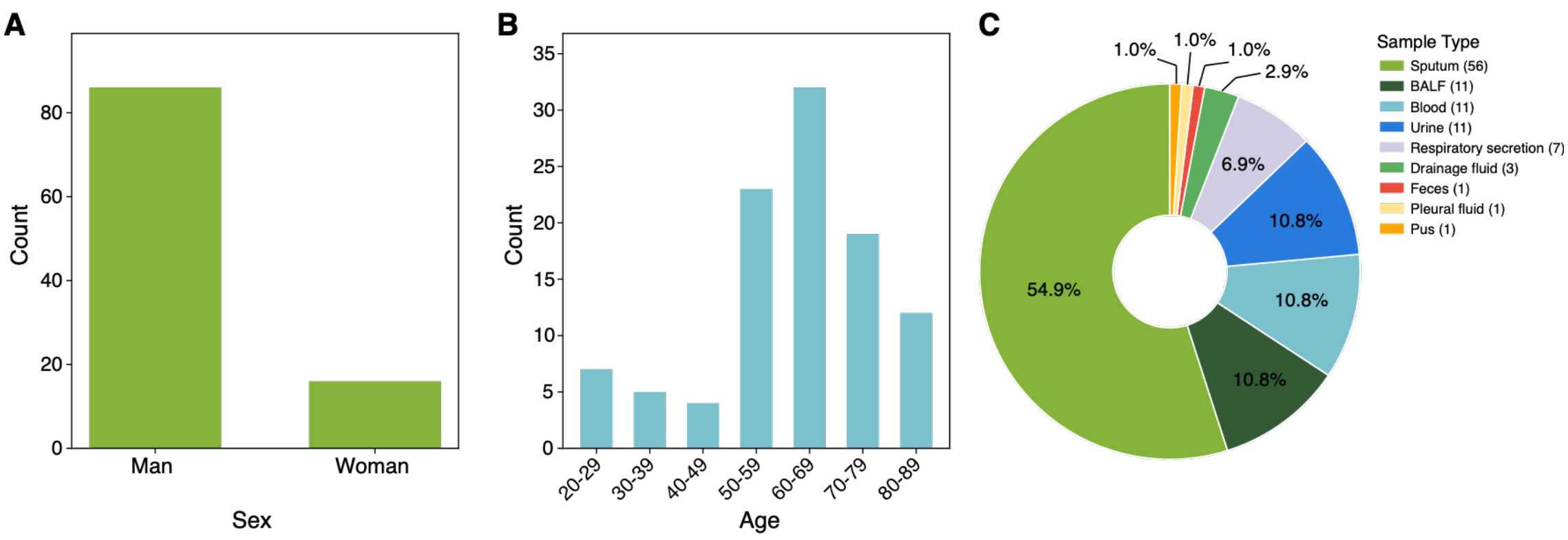

Fig. S2. Geographic origin of 4,262 public *K. pneumoniae* genomes analyzed in this study. Countries with the highest representation are highlighted.

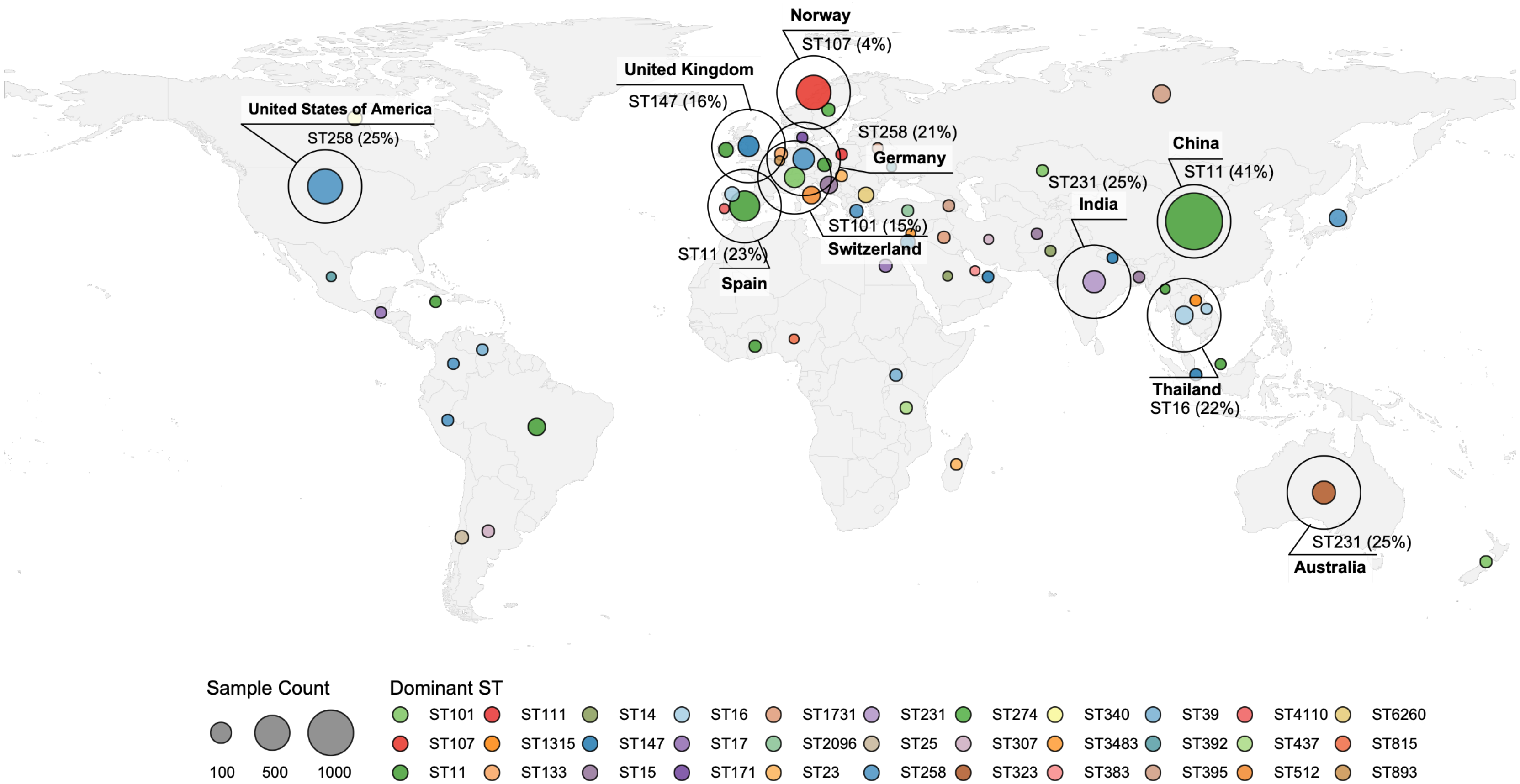

Fig. S3. Pangenome composition and functional landscape of *K. pneumoniae* (n=1880). (A) Proportional distribution of orthologous groups (OGs) across pangenome partitions (core, soft-core, shell, and cloud). (B) Total gene abundance contributed by each pangenome partition across all genomes. (C) KEGG pathway enrichment across different pangenome regions.

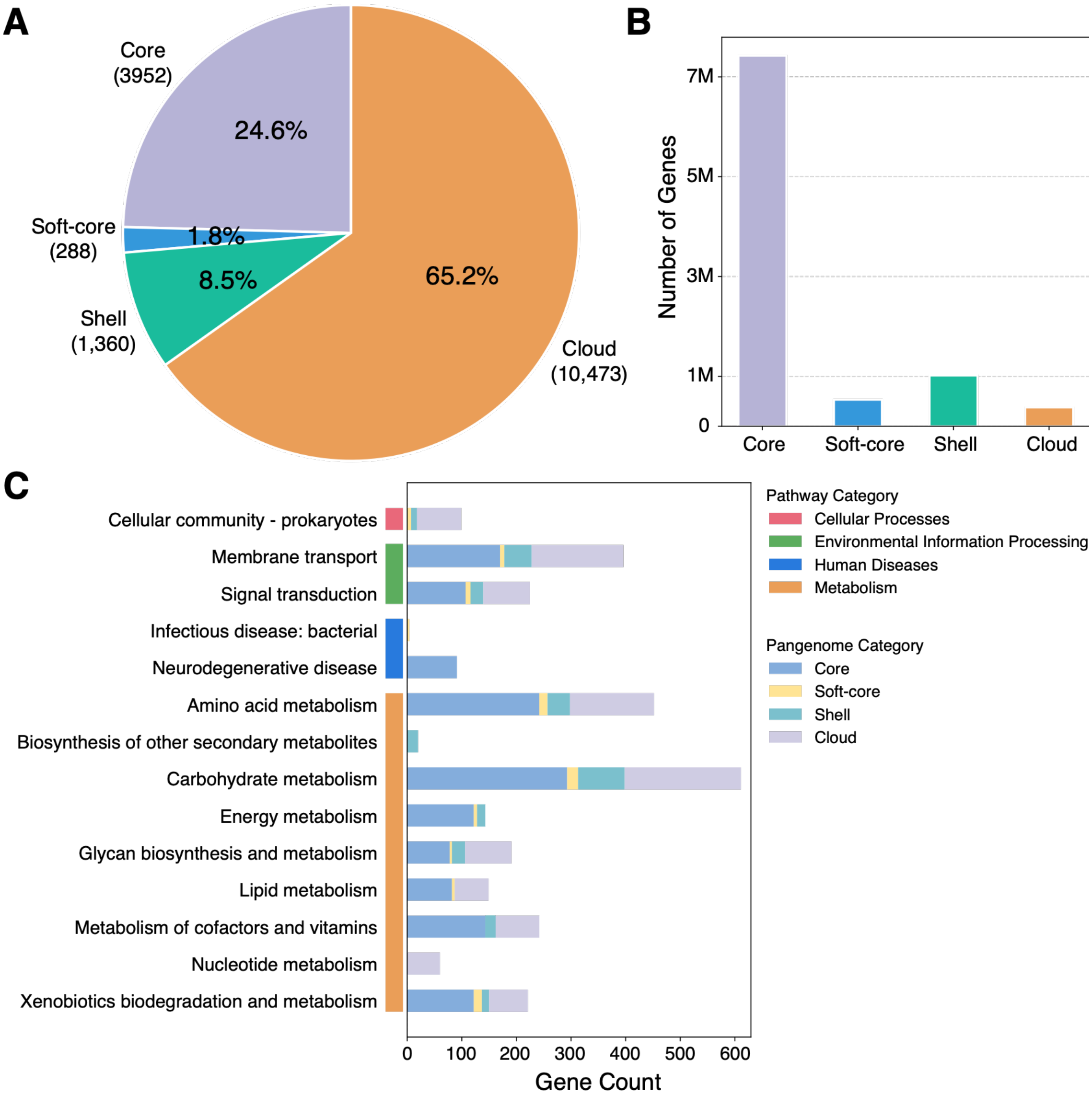

Fig S4. Genomic relatedness of chromosome sequences assessed by ANI and alignment fraction. (A) Distribution of pairwise average nucleotide identity (ANI). (B) Distribution of pairwise alignment fraction (AF).

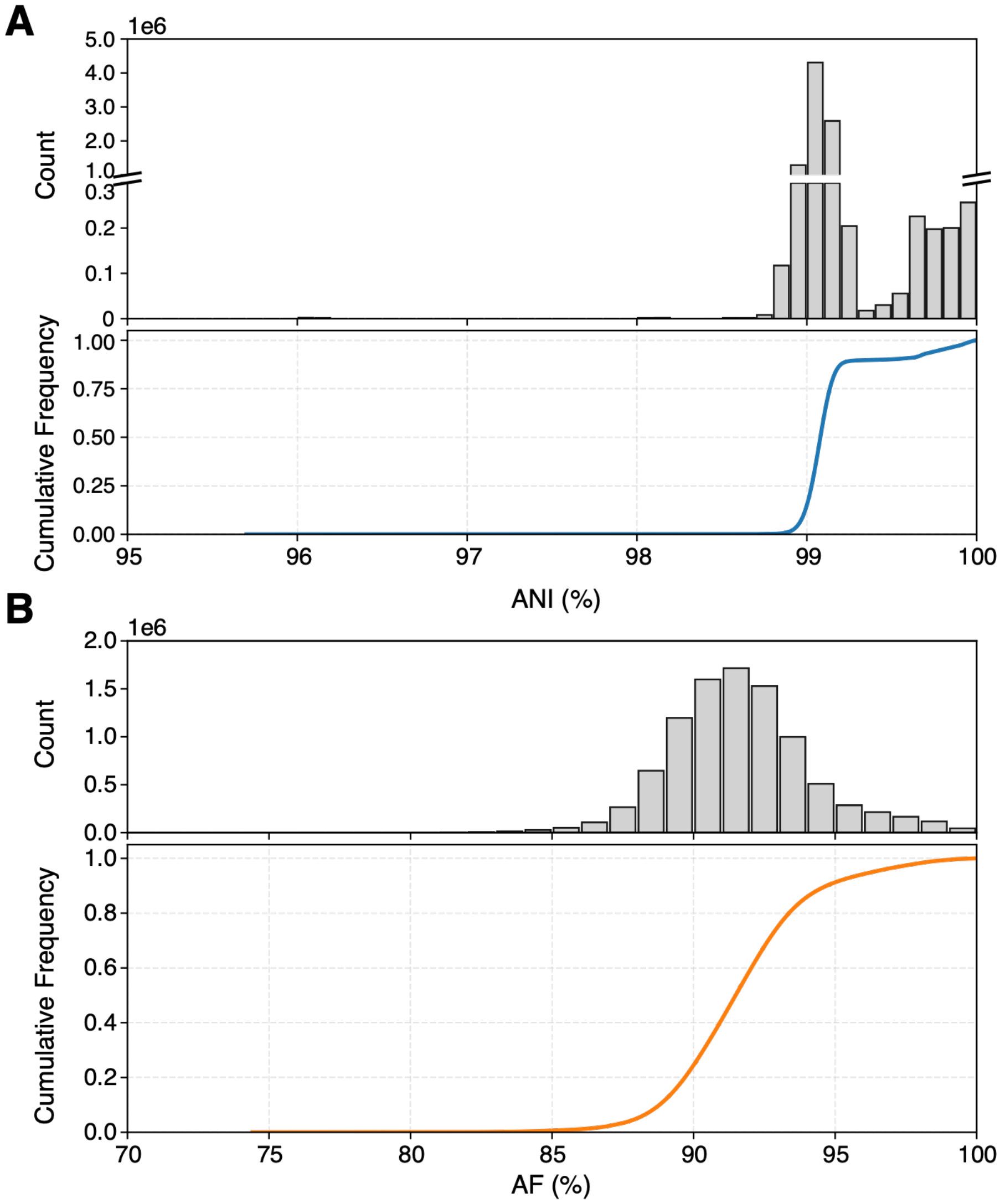

Fig S5. Genomic relatedness of plasmid sequences assessed by ANI and alignment fraction. (A) Distribution of pairwise average nucleotide identity (ANI). (B) Distribution of pairwise alignment fraction (AF).

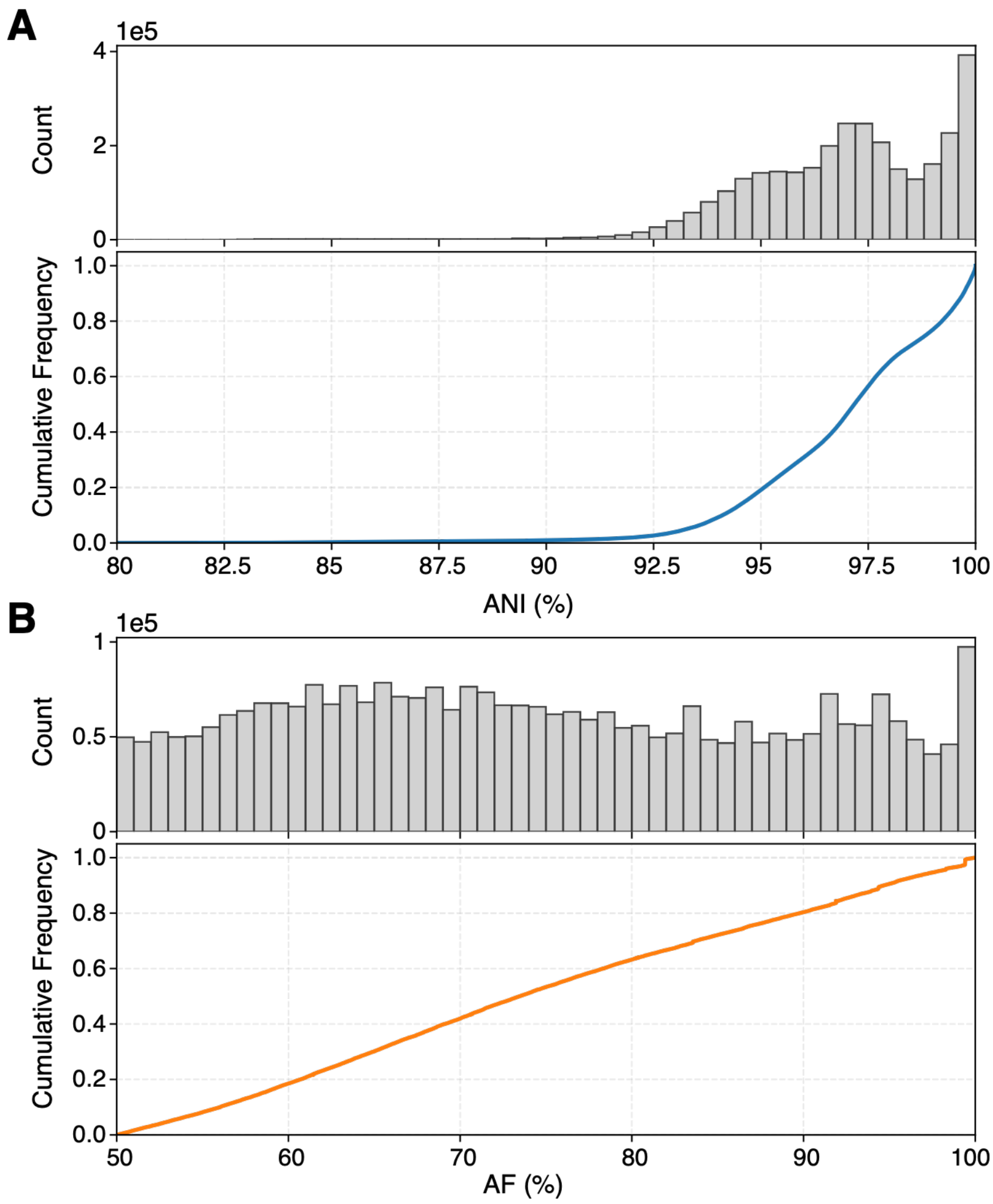

Fig. S6. Chromosomal clustering threshold determination based on ANI analysis. The number of detected communities rises steeply between 99.0% and 99.4% ANI and plateaus above 99.5%.

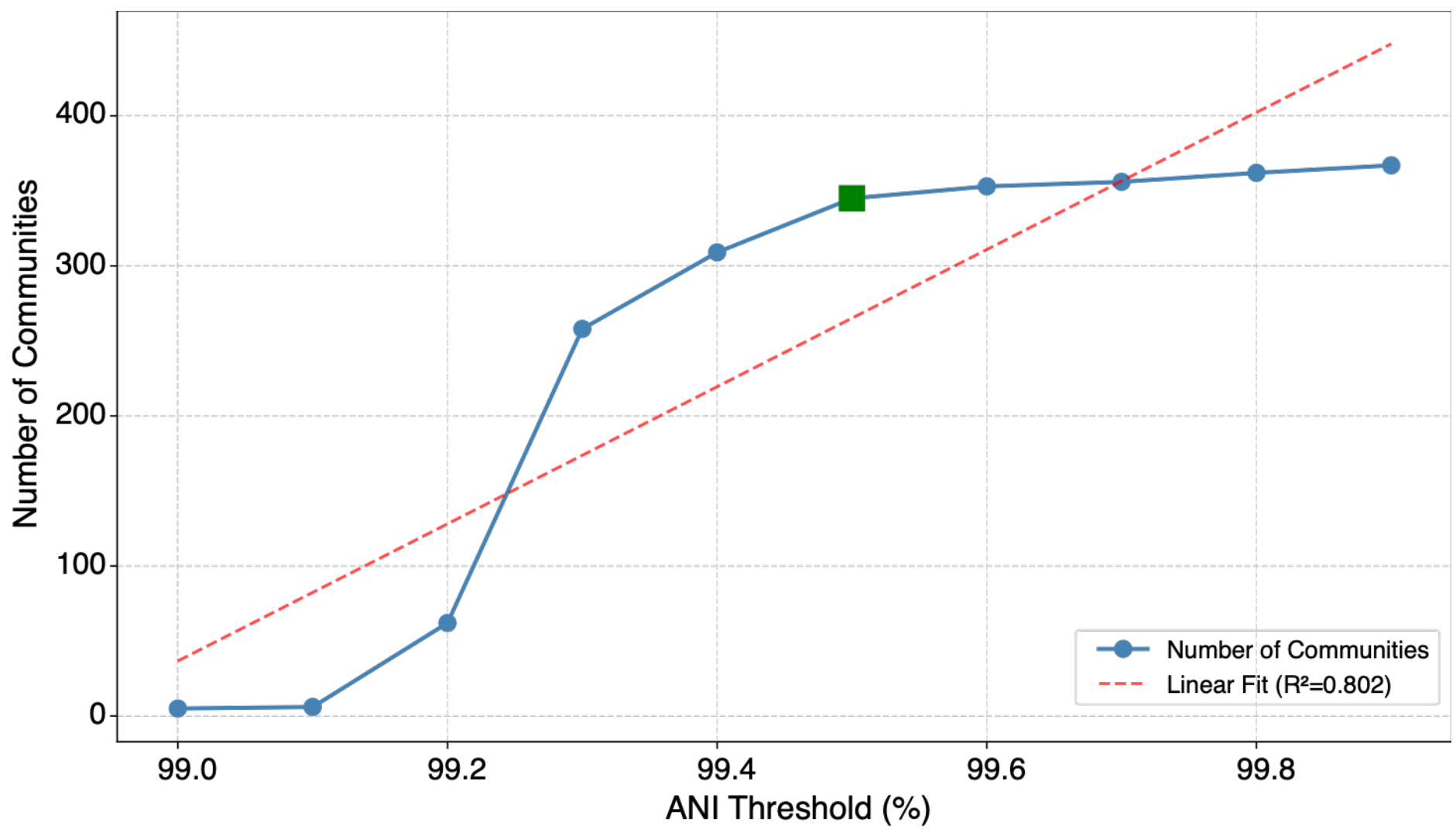

Fig. S7. Plasmid size distribution across the 15 largest plasmid communities. Boxplots with all individual data points overlaid show the median and IQR for each community.

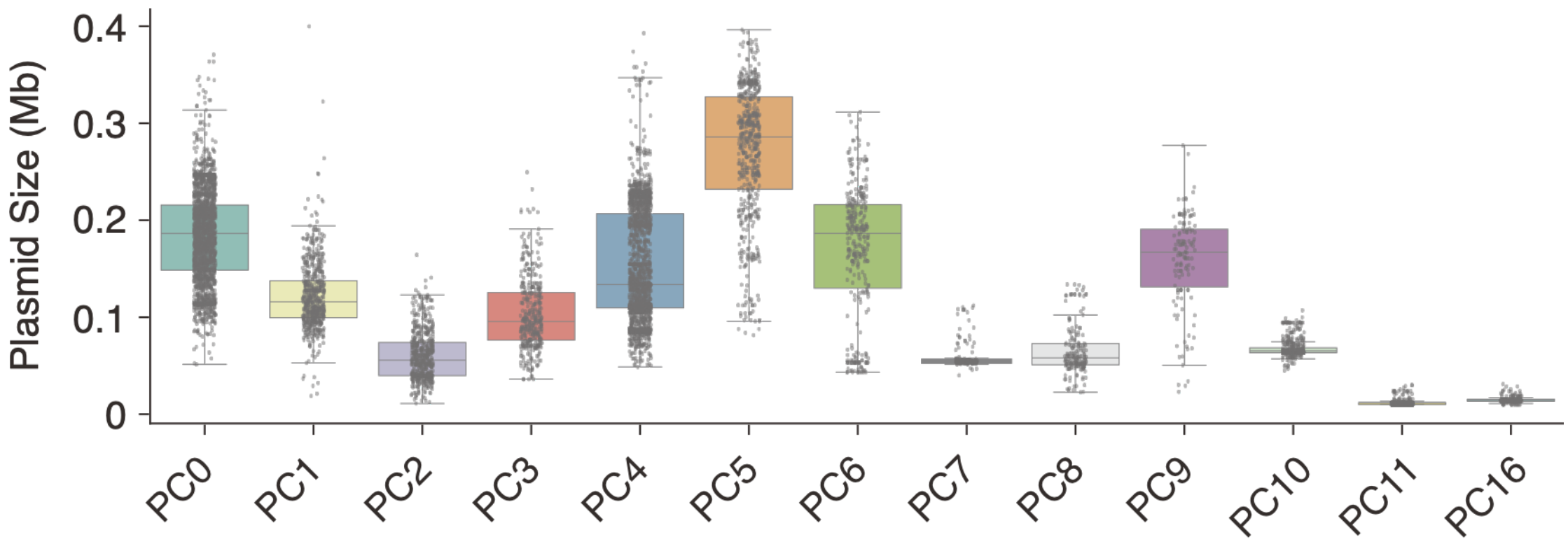

Fig. S8. Association patterns of plasmid communities with replicon types, host STs, and geographic origin. (A) Plasmid community association with replicon types. (B) Association with host sequence types (STs). (C) Association with geographic origin.

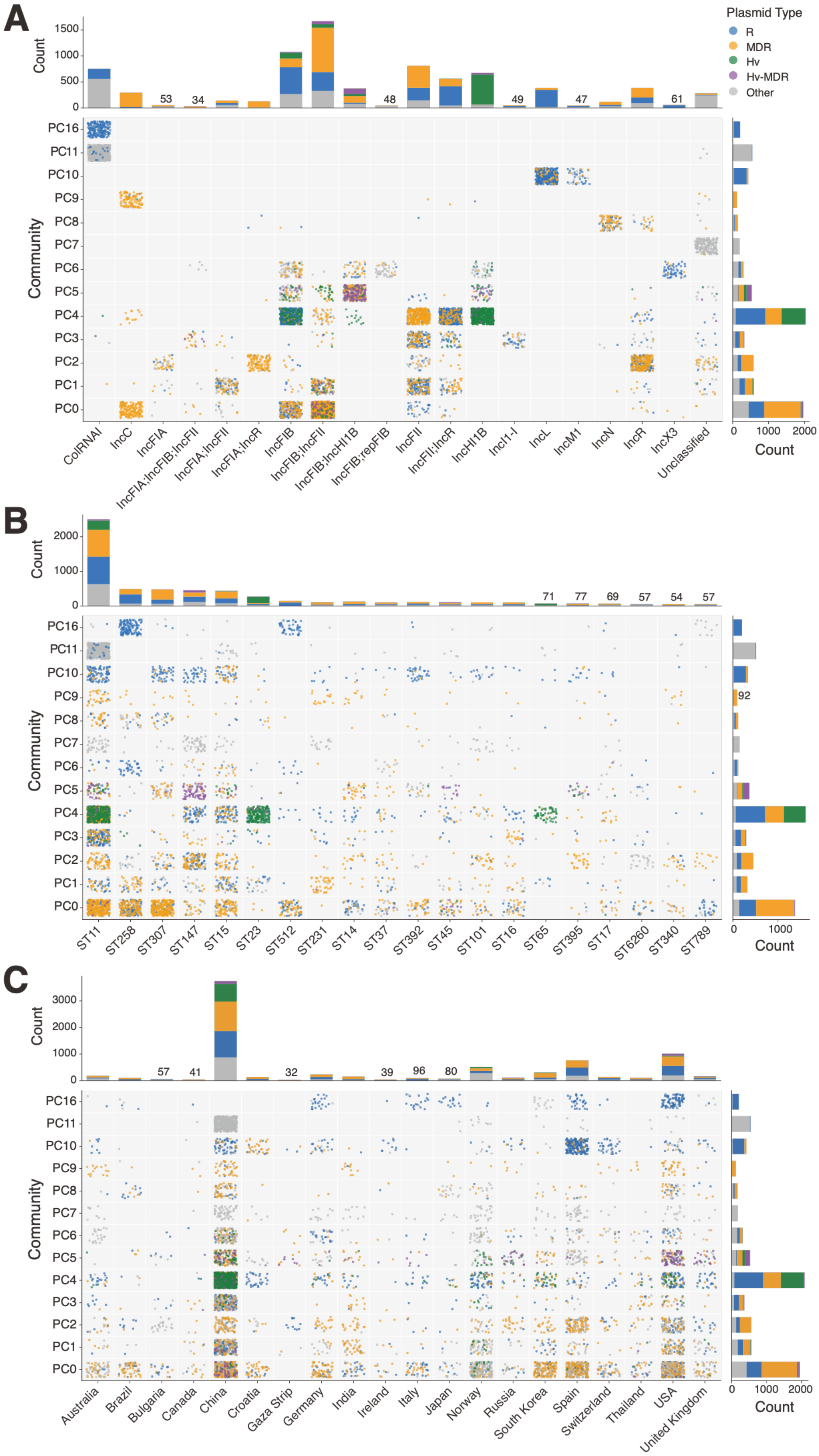

Fig. S9. Distribution patterns of carbapenemase-carrying plasmids across plasmid communities. (A) Association with replicon types. (B) Association with host sequence types (STs). (C) Association with geographic origin.

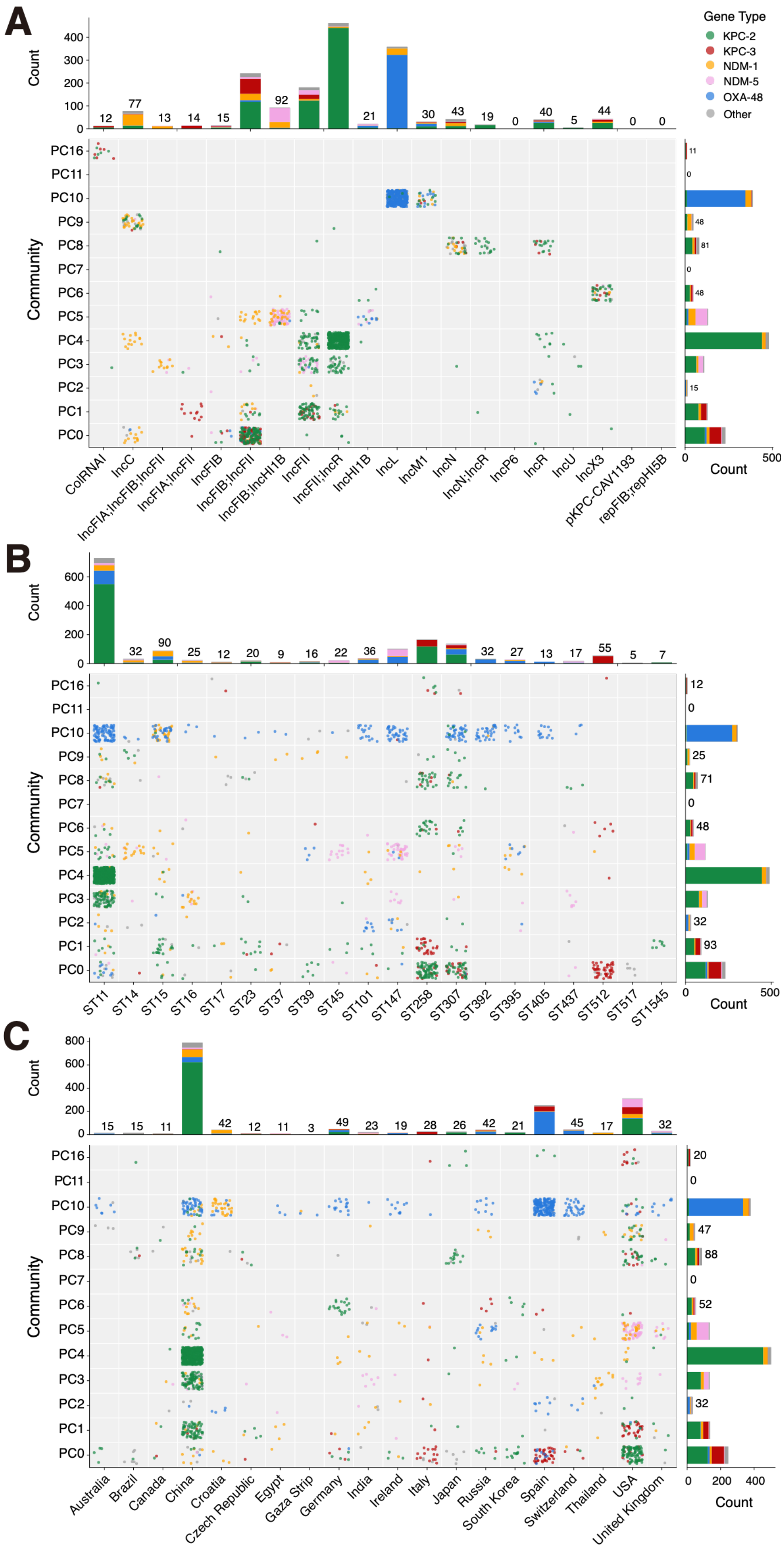

Fig. S10. Bipartite network linking chromosomal communities (ChrC) and plasmid communities (PC). Edge width is proportional to the number of plasmids associated with each chromosomal lineage.

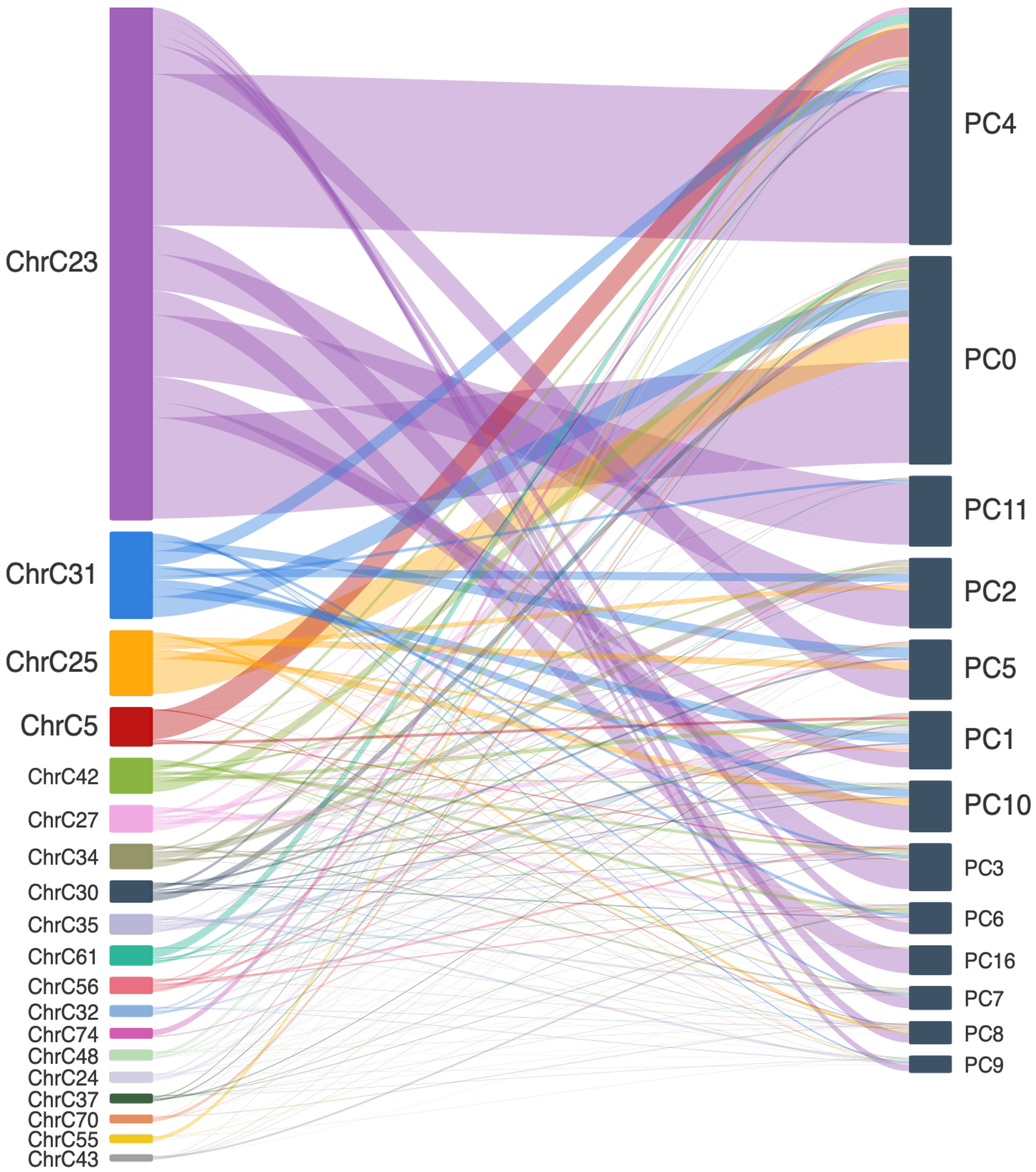

Fig. S11. Phylogeny of plasmid and chromosomal virulence genes in *K. pneumoniae*. (A) Plasmid-encoded aerobactin cluster (*iucABCD-iutA*). (B–F) Chromosomal cluster or gene: (B) *yagV–K*, (C) *entAB*, (D) *fepC*, (E) *omp*, (F) *ybt* (yersiniabactin).

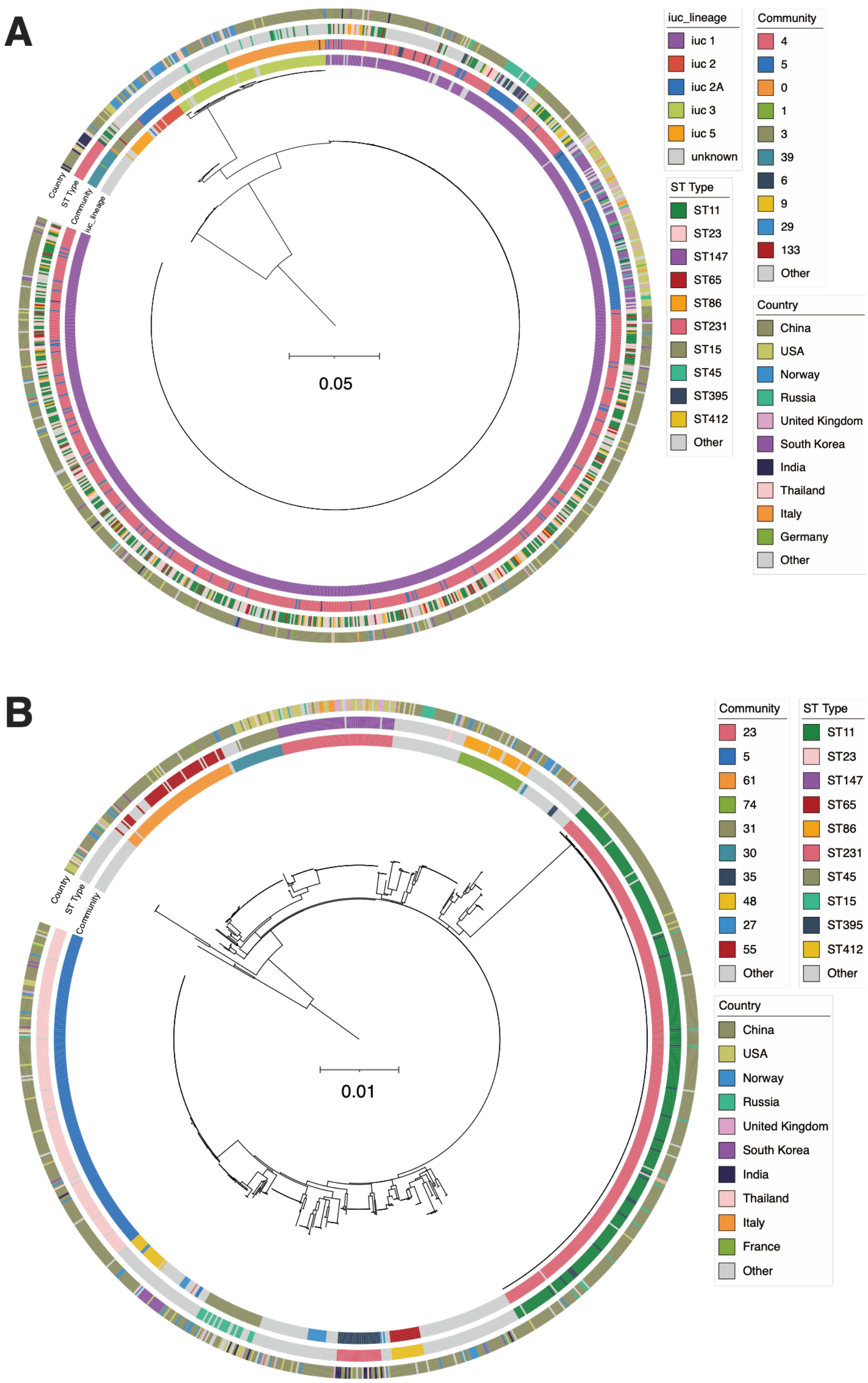

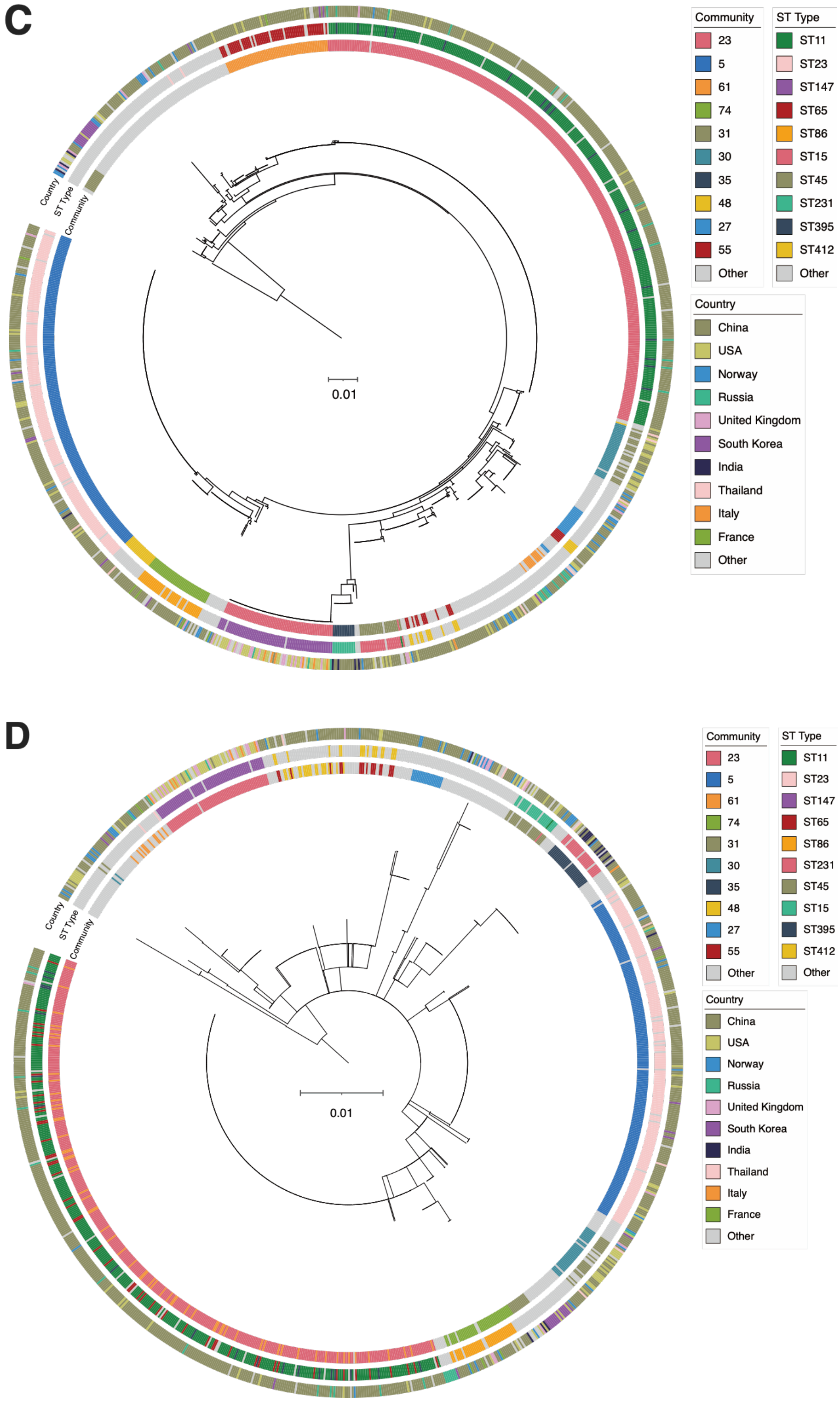

C
Country
ST Type
Community
0.01
Community
23
5
61
74
31
30
35
48
27
55
Other
ST Type
ST11
ST23
ST147
ST65
ST86
ST15
ST45
ST231
ST395
ST412
Other
Country
China
USA
Norway
Russia
United Kingdom
South Korea
India
Thailand
Italy
France
Other
D
Country
ST Type
Community
0.01
Community
23
5
61
74
31
30
35
48
27
55
Other
ST Type
ST11
ST23
ST147
ST65
ST86
ST231
ST45
ST15
ST395
ST412
Other
Country
China
USA
Norway
Russia
United Kingdom
South Korea
India
Thailand
Italy
France
Other

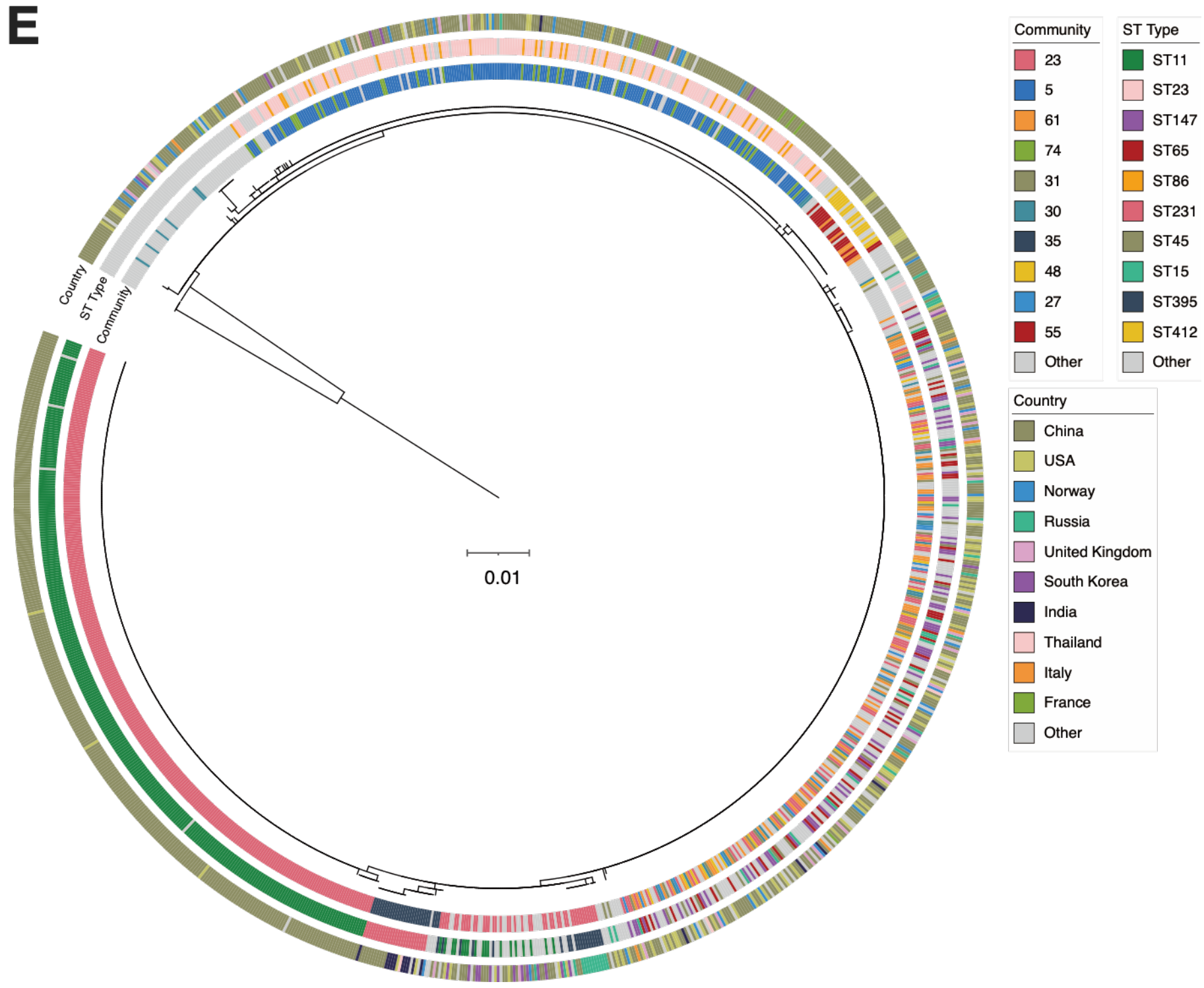
E
Country
ST Type
Community
0.01
Community
23
5
61
74
31
30
35
48
27
55
Other
ST Type
ST11
ST23
ST147
ST65
ST86
ST231
ST45
ST15
ST395
ST412
Other
Country
China
USA
Norway
Russia
United Kingdom
South Korea
India
Thailand
Italy
France
Other

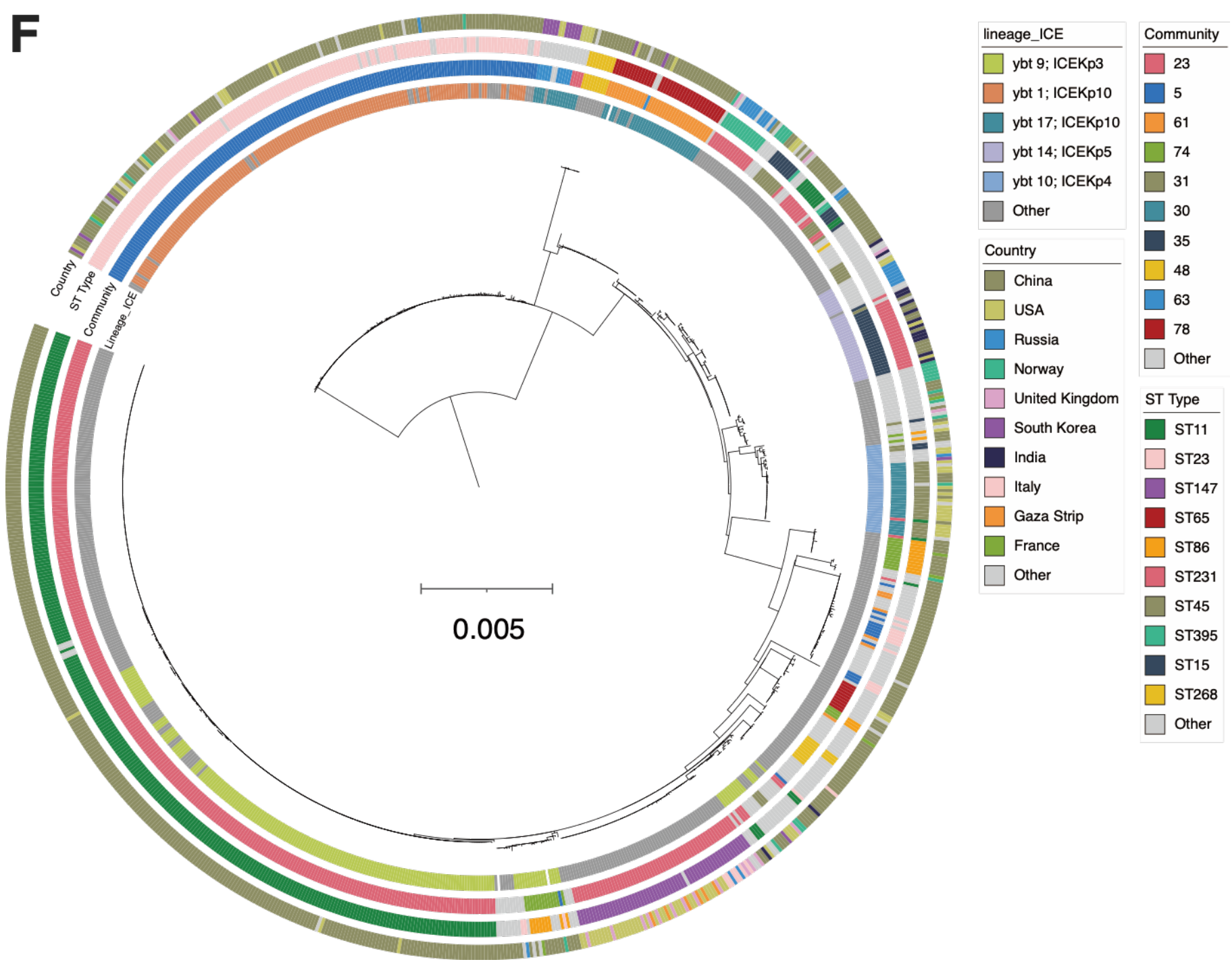
F
Country
ST Type
Community
Lineage_ICE
0.005
lineage_ICE
ybt 9; ICEKp3
ybt 1; ICEKp10
ybt 17; ICEKp10
ybt 14; ICEKp5
ybt 10; ICEKp4
Other
Country
China
USA
Russia
Norway
United Kingdom
South Korea
India
Italy
Gaza Strip
France
Other
Community
23
5
61
74
31
30
35
48
63
78
Other
ST Type
ST11
ST23
ST147
ST65
ST86
ST231
ST45
ST395
ST15
ST268
Other

Fig. S12. Phylogenetic analysis of antimicrobial resistance genes in *Klebsiella pneumoniae*. (A) Plasmid-encoded carbapenemase genes. (B) Chromosomal β-lactamase genes. (C) Chromosomal *fosA* gene.

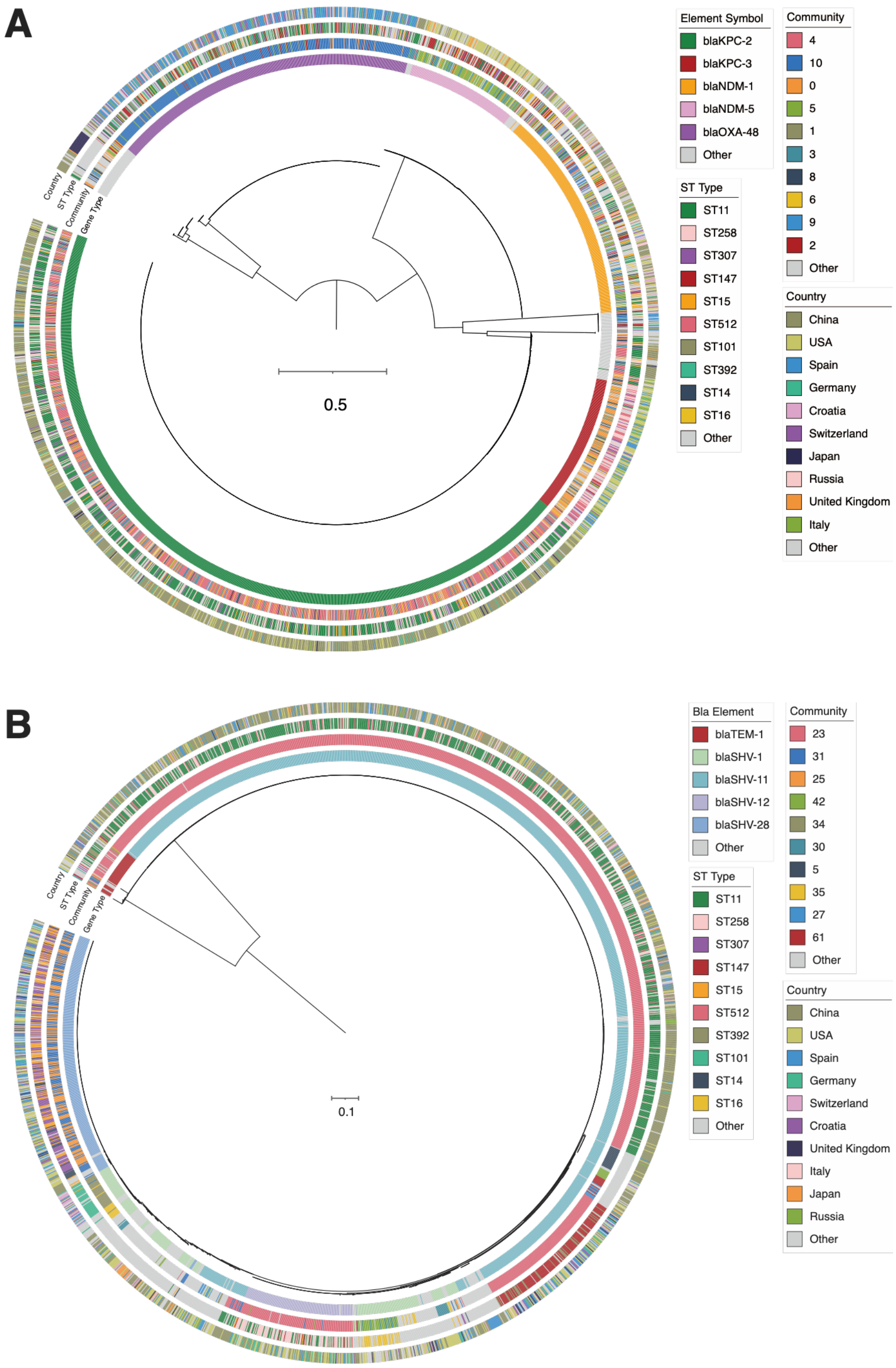

C

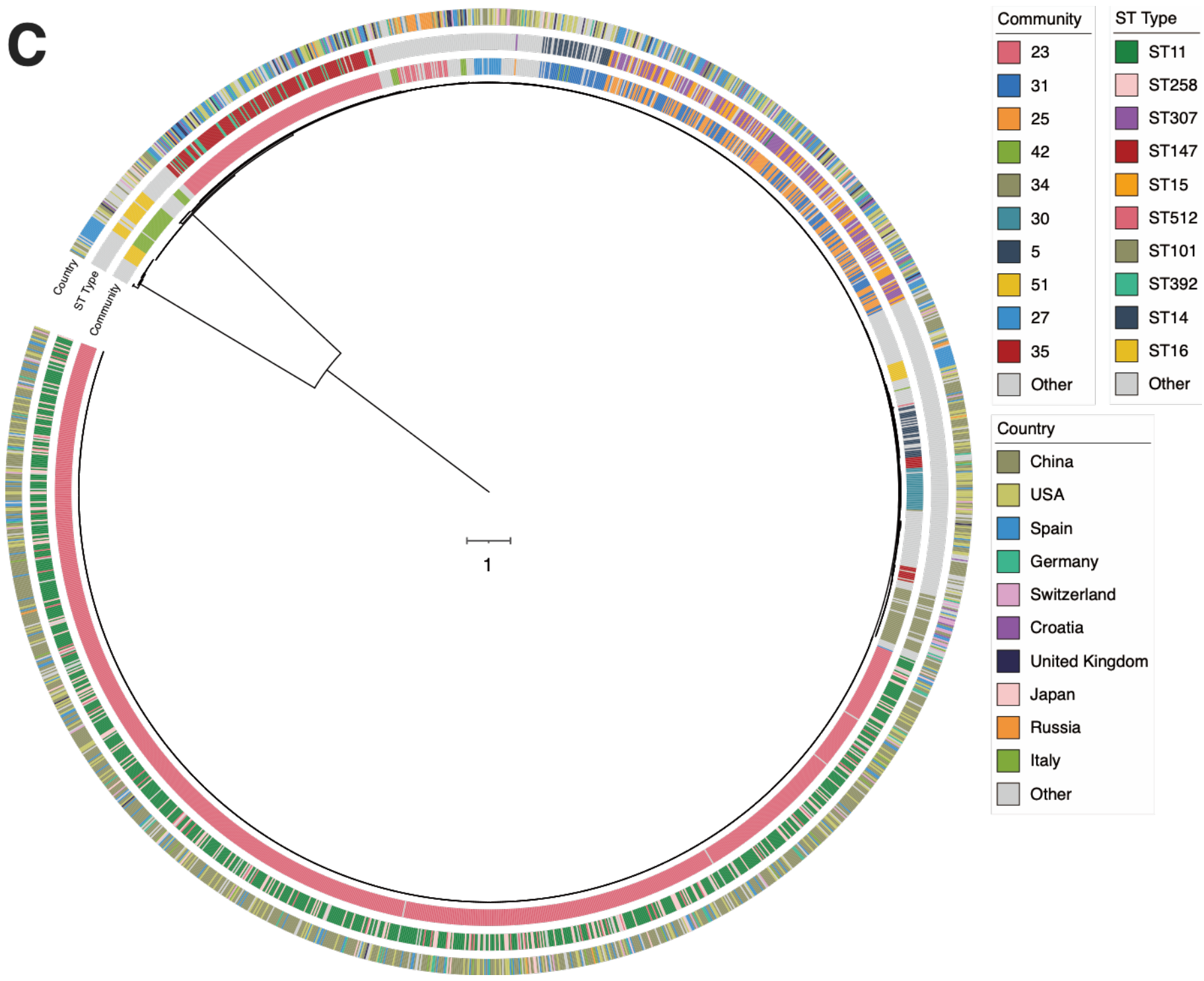
Country
ST Type
Community
1
Community
23
31
25
42
34
30
5
51
27
35
Other
ST Type
ST11
ST258
ST307
ST147
ST15
ST512
ST101
ST392
ST14
ST16
Other
Country
China
USA
Spain
Germany
Switzerland
Croatia
United Kingdom
Japan
Russia
Italy
Other

Fig. S13. Length distribution of prophages by genomic context and completeness category. (A) Chromosomal prophages. (B) Plasmid-associated prophages. Boxplots show median, IQR, and outliers for intact, incomplete, and questionable prophages.

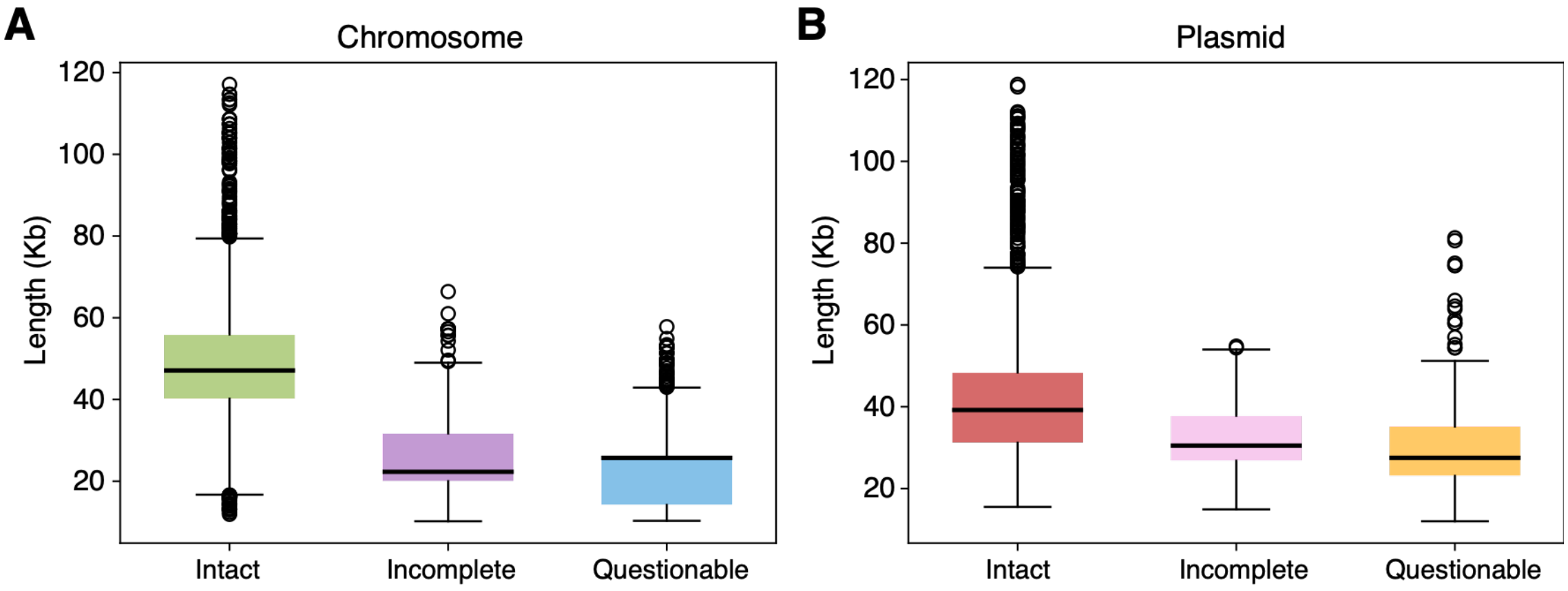

Fig. S14. Pairwise sequence similarity among prophage sequences. (A) Distribution of average nucleotide identity (ANI). (B) Distribution of alignment fraction (AF).

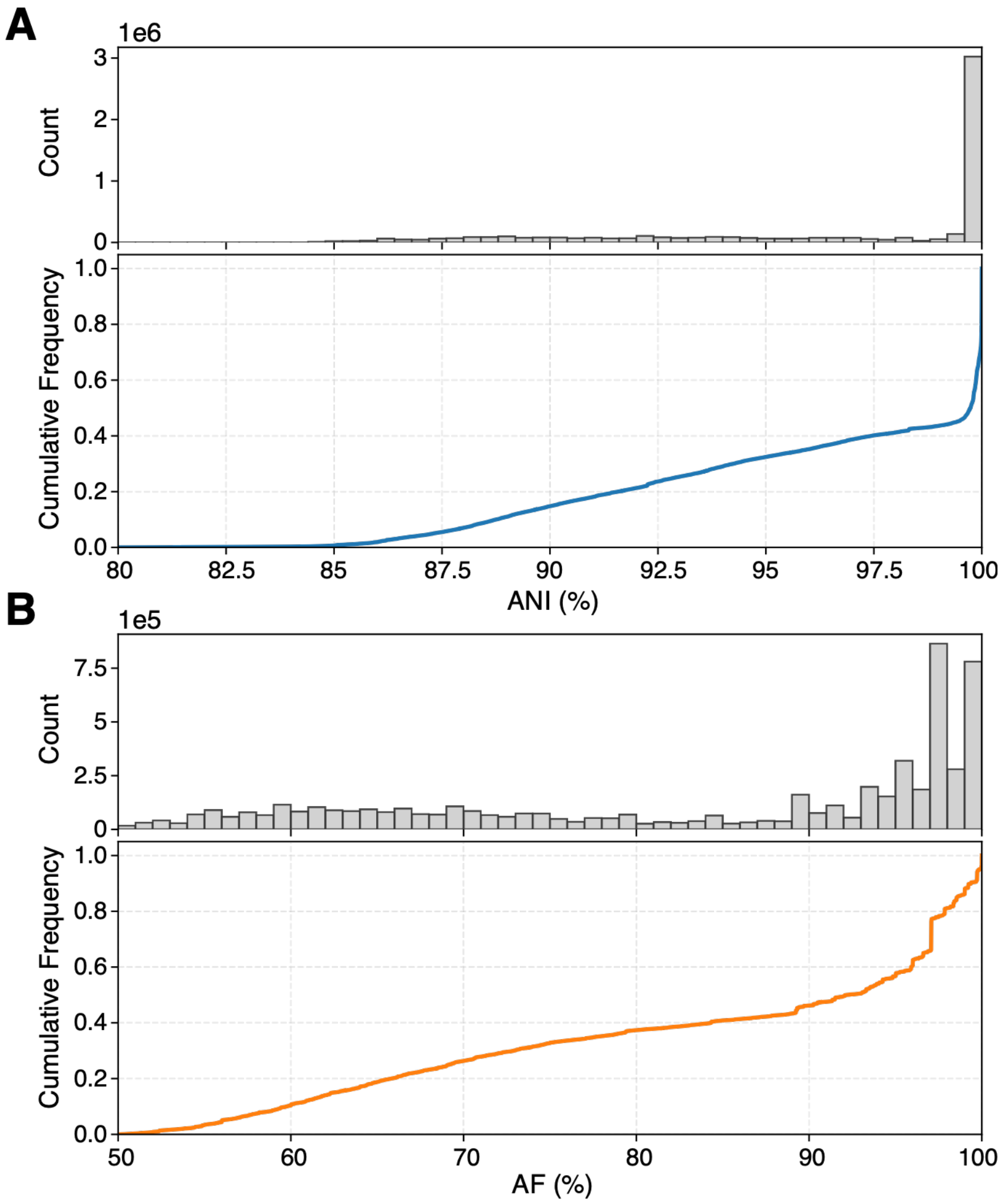

Fig. S15. Distribution of resistance gene-carrying prophages by sequence type and geographic origin. (A) Distribution across sequence types (STs), with colors indicating resistance gene classes. (B) Geographic distribution across countries.

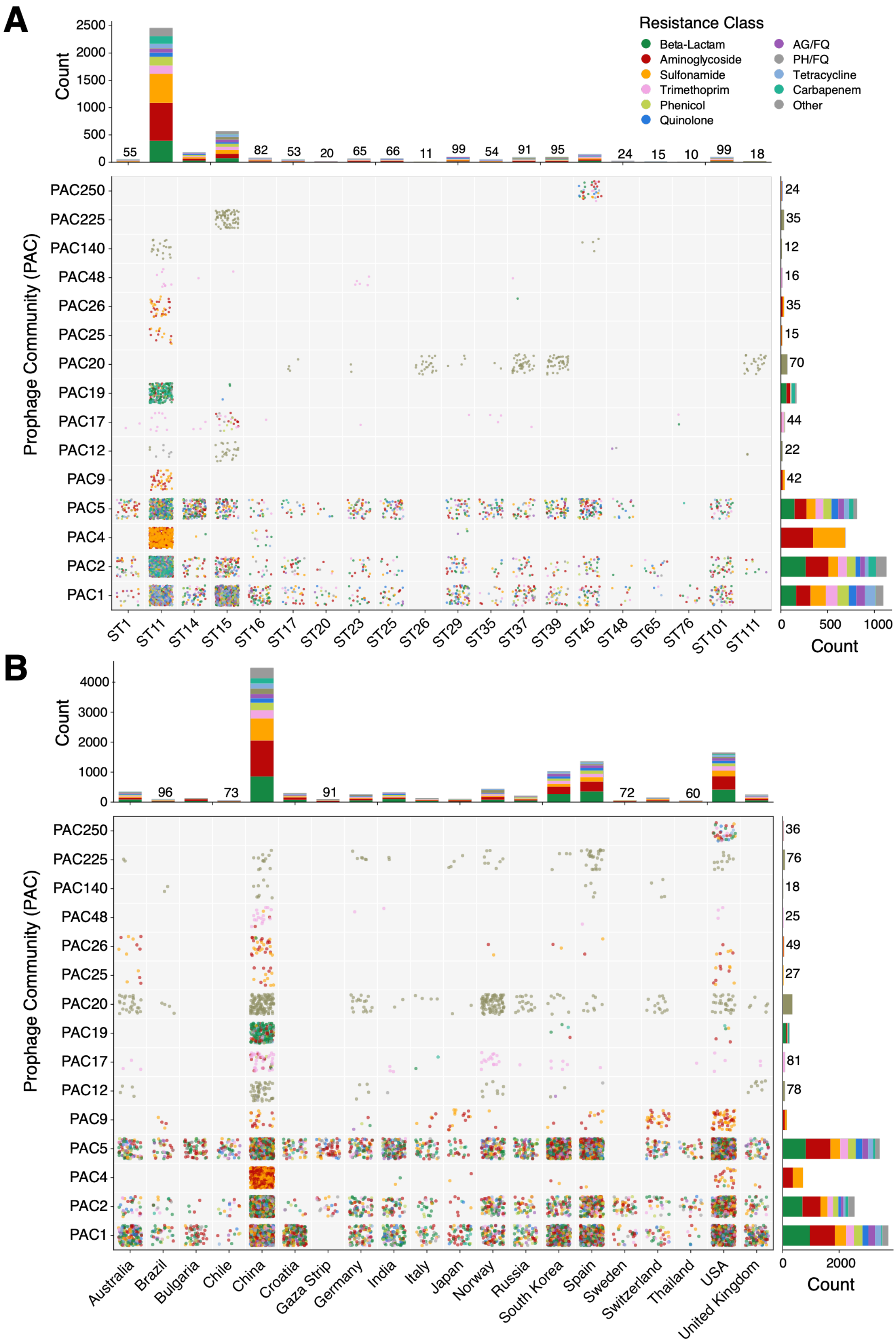

Fig. S16. Multidimensional characterization of plasmid communities in Qujing *K. pneumoniae* isolates. Panels show (from left to right): sequence type (ST) distribution (color intensity indicates proportion), antimicrobial resistance gene classes, virulence gene profiles, plasmid size distribution, and plasmid count per community.

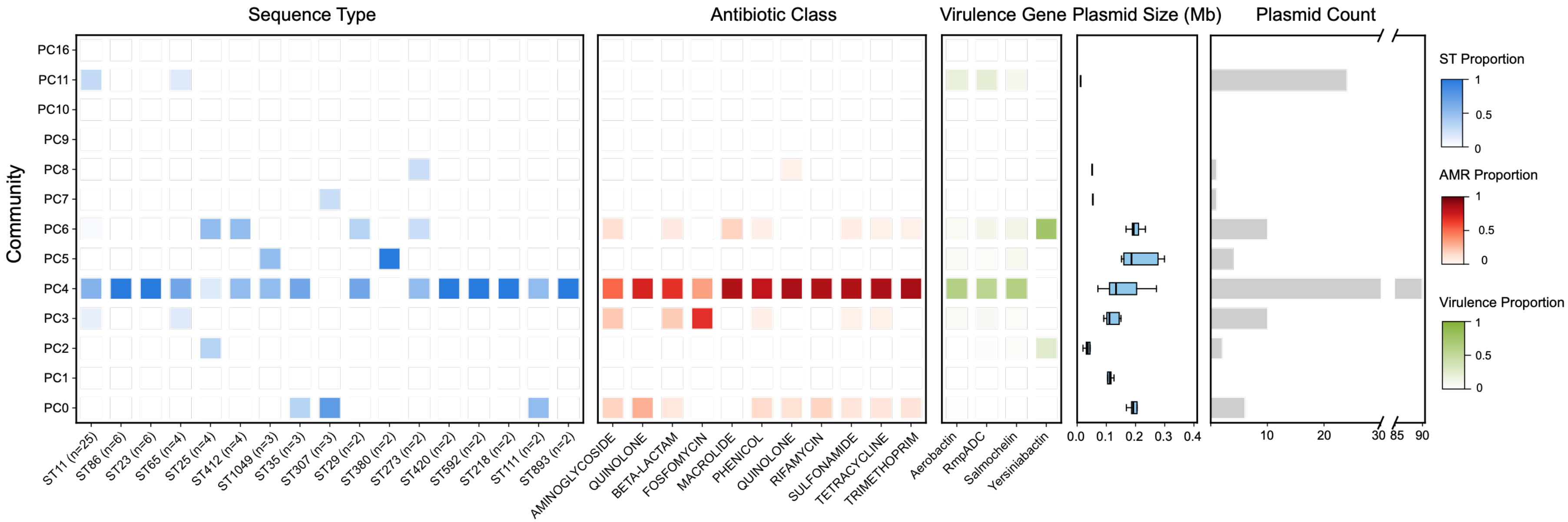

Fig. S17. Circular maps of Hv-MDR plasmids from KP3-8-2 (A) and KP3-20-2 (B), both belonging to the PC0 lineage (~250 kb each). From inner to outer: size scale (kb), negative GC skew, positive GC skew, GC content, and annotated genes.

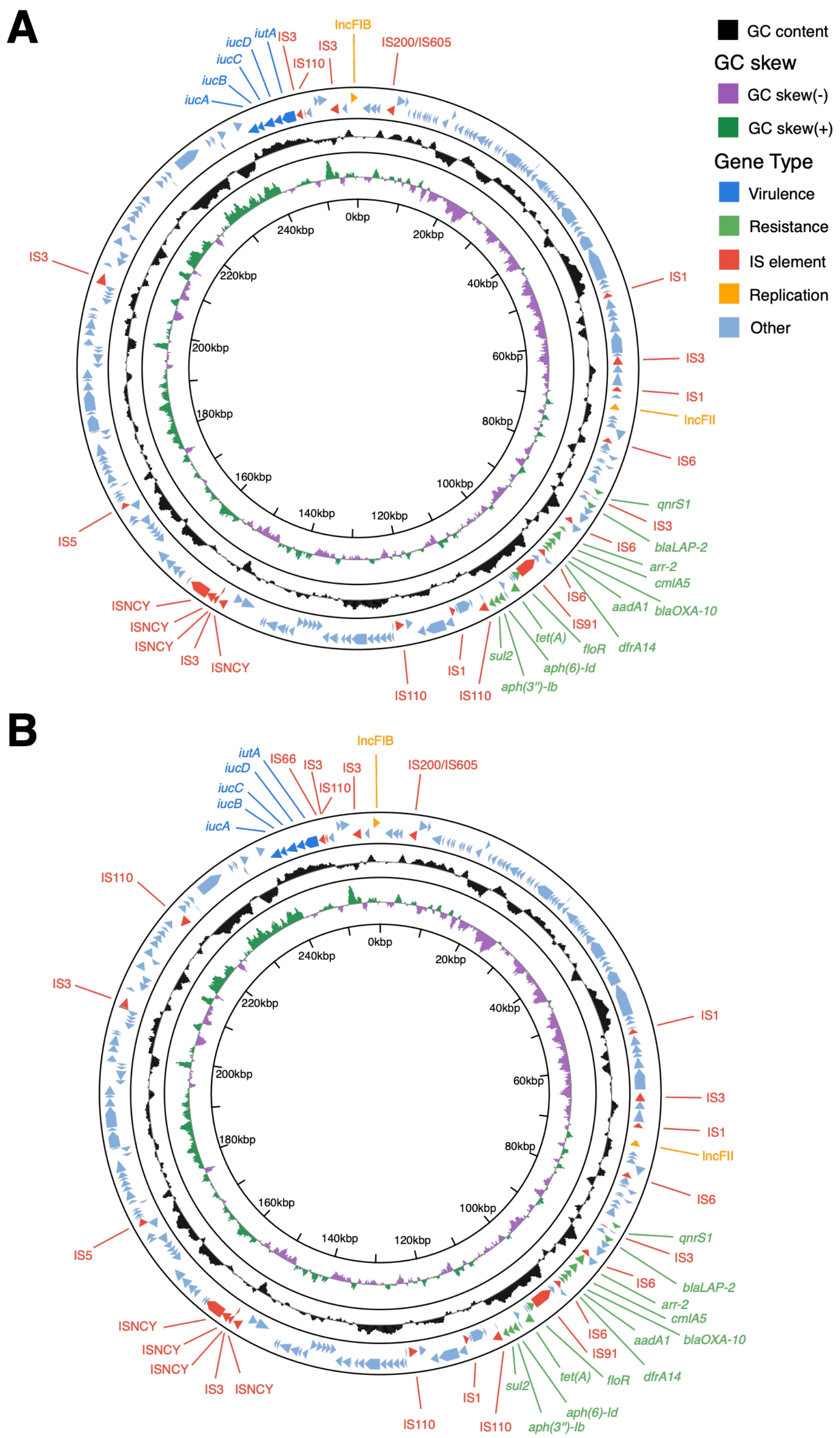

Supplement: Supplementary file 1 [file Data_Sheet_1.pdf]
